# Interfacial pH Dynamics in Lattice-Nitrogen Protonation to Ammonia

**DOI:** 10.1021/jacs.6c14168

**Published:** 2026-10-07

**Authors:** David Kumar Yesudoss, Joshua Ferguson, Anabela Meier, Abdoulaye Djire

**Affiliations:** † Artie McFerrin Department of Chemical Engineering, 14736 Texas A&M University, College Station, Texas 77843, United States; ‡ Department of Materials Science and Engineering, Texas A&M University, College Station, Texas 77843, United States; § Department of Chemistry, Texas A&M University, College Station, Texas 77843, United States

## Abstract

We recently discovered a new class of nitride MXenes (MNenes) that undergo lattice-nitrogen protonation to form ammonia under cathodic potentials. Unexpectedly, neutral electrolytes outperform both acidic and alkaline media, revealing the critical role of the interfacial proton environment in the formation of ammonia. However, how dynamic interfacial pH governs this mechanism remains poorly understood, because conventional ex situ characterization cannot probe local pH under operating conditions. Here, we develop an in-house in situ attenuated total reflection Fourier transform infrared (ATR-FTIR) spectroscopy with phosphate speciation as a vibrational pH reporter to monitor in real time the interfacial pH near the Ti_2_NT_*x*_ MNene catalyst during cathodic polarization. On one hand, we found that in a nominally neutral phosphate buffer, cathodic operation drives the Ti_2_NT_*x*_ interface strongly alkaline despite the fact that the bulk remains neutral, whereas a mildly acidic electrolyte partially moderates this pH rise by increasing proton availability. On the other hand, a strongly acidic electrolyte minimizes interfacial pH changes but can also promote non-catalytic lattice-nitrogen protonation and release of NH_3_. Time-dependent measurements show that interfacial pH is governed by the balance between current-driven alkalinity generation and electrolyte-mediated neutralization. Overall, this in situ study shows that electrolyte pH and current density must be tuned together to maintain a fairly neutral interfacial environment favorable for lattice-nitrogen protonation to ammonia.

## Graphical abstract

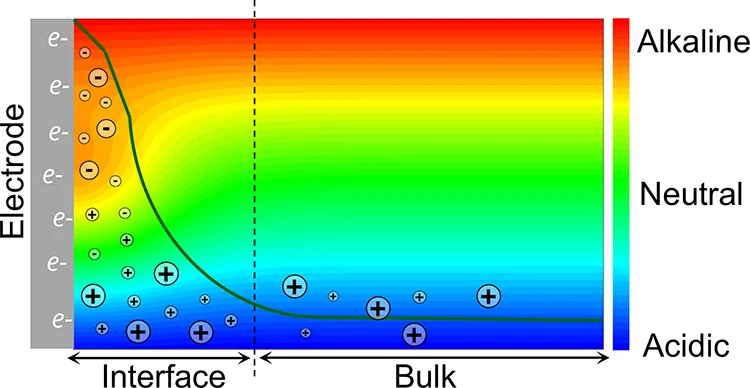

## Introduction

Ammonia is one of the most important chemicals in modern society, serving as the foundation for fertilizer production, nitrogen-containing chemicals, and emerging carbon-free energy carriers.^1,2^ Industrial NH_3_ synthesis is currently dominated by the Haber-Bosch process, which enabled global food production but remains energy-intensive and closely tied to fossil-derived hydrogen (H_2_).^1–3^ As renewable electricity becomes increasingly available, the electrochemical nitrogen reduction reaction (NRR) has attracted significant interest as a possible route to distributed NH_3_ synthesis under ambient conditions.^3–5^ In principle, NRR can couple N_2_ from air with protons and electrons from water to produce NH_3_ without the high temperature, high pressure, and centralized infrastructure required by Haber-Bosch chemistry. However, translating this into a practical aqueous electrochemical process remains difficult because N_2_ activation is kinetically demanding, N_2_ solubility in water is low, and the hydrogen evolution reaction (HER) competes strongly for the same protons and electrons.^4–7^ Most aqueous NRR catalysts are discussed through associative or dissociative N_2_ activation pathways, where molecular N_2_ adsorbs onto a surface site and is progressively protonated to form NH_3_.^4,5^ These pathways face a fundamental selectivity challenge because the same active sites that bind and activate N_2_ often also promote proton reduction to form H_2_.^4–6^ Hence, many reported aqueous NRR systems operate in low-yield regimes where trace nitrogen-containing impurities, ubiquitous NH_3_ contamination, or catalyst instability can obscure the true origin of NH_3_.^6,8–10^ Therefore, identifying the origin of NH_3_ in aqueous electrochemical systems remains challenging, particularly when nitrogen-containing electrode materials are used. Because these materials contain nitrogen within their crystal lattice, the detected NH_3_ may originate either from the reduction of externally supplied N_2_ or from protonation and removal of nitrogen already present in the catalyst lattice.^10–12^

Previous studies on Mo_2_N have shown that NH_3_ detected during electrochemical measurements can originate from lattice nitrogen rather than gaseous N_2_ reduction and that the extent of nitrogen release depends strongly on the surrounding electrolyte environment.^10^ These results indicate that lattice N protonation is highly sensitive to local chemical conditions and that NH_3_ formation from nitrogen-containing materials cannot be interpreted from the bulk electrolyte composition alone. In lattice-nitrogen-mediated (LNM) pathways proposed for nitrogen chemistry, this lattice-N protonation step is exploited as part of a vacancy-mediated reaction sequence.^12–16^ Regardless of whether subsequent vacancy replenishment occurs, the initial protonation of lattice nitrogen is therefore expected to depend strongly on the proton environment experienced by the nitride surface.

Ti_2_NT_*x*_ MNene provides a useful platform to investigate this question because its lattice nitrogen can undergo protonation under electrochemical conditions and the release of lattice nitrogen can also depend strongly on electrolyte pH. Our previous work established Ti_2_NT_*x*_ as a nitrogen-containing electrochemical material in which lattice nitrogen can participate in NH_3_-forming chemistry.^16^ However, the local proton environment experienced by Ti_2_NT_*x*_ during the cathodic polarization has not been directly measured. This is important because the proton availability required for lattice N protonation is not simply determined by the initial bulk pH. Proton-consuming reductive processes, particularly HER, can continuously deplete proton donors or generate OH^–^ near the electrode surface, producing an interfacial environment that is substantially more alkaline than the surrounding bulk electrolyte.^17–19^ However, whether such interfacial pH changes occur on Ti_2_NT_*x*_ during reductive polarization and how they influence lattice-nitrogen protonation remain unknown.^20^ If the interface becomes too alkaline, the availability of proton donors for lattice-nitrogen protonation may become limited. On the other hand, strongly acidic conditions can promote chemical protonation and release of lattice nitrogen even in the absence of an applied potential. Therefore, understanding lattice N reactivity requires distinguishing the static chemical effect of bulk pH from the dynamically evolving proton environment generated during electrochemical operation.

Recently, the interfacial pH has been increasingly recognized as a critical hidden variable in aqueous electrocatalysis. Because heterogeneous electrochemical reactions occur at the electrode-electrolyte boundary, the concentrations of H^+^, OH^–^, buffer species, reactants, and products can deviate strongly from the bulk electrolyte. These deviations affect reaction rates, selectivity, catalyst stability, and even the correct assignment of the electrode potential on the RHE scale. Several methods have been developed to probe local pH, including scanning probe techniques, fluorescence microscopy, rotating ring-disc electrode approaches, and vibrational spectroscopy.^17–19,21^ Among these, attenuated total reflectance surface-enhanced infrared absorption spectroscopy (ATR-SEIRAS) provides a powerful approach because it can monitor buffer speciation within the near-electrode region under operating conditions. Tarpeh and co-workers recently showed that phosphate buffer speciation can be used as an infrared reporter of interfacial pH during nitrate reduction on Cu.^21^ They demonstrated that the interfacial pH can increase dramatically during cathodic polarization even when the bulk pH remains unchanged, highlighting the difficulty of assuming that the bulk pH represents the reaction environment.

Inspired by this concept, the present work applies phosphate-based interfacial pH measurements to a nitrogen-containing Ti_2_NT_*x*_ electrode and focuses on how the interfacial proton environment evolves as a function of the applied potential, time, and bulk proton availability. In particular, we show that cathodic alkalization becomes stronger with an increasing reductive current. This local pH increase can be substantially moderated by increasing the proton reservoir in the bulk electrolyte; however, it is not necessarily eliminated. Phosphate is well-suited for this purpose because its protonation state changes predictably across acidic, neutral, and basic pH regimes, and each phosphate species exhibits distinct infrared-active P–O vibrational features.^21,22^ By calibrating the pH-dependent spectral response of H_3_PO_4_, H_2_PO_4_^–^, HPO_4_^2–^, and PO_4_^3–^, the interfacial phosphate speciation can be translated into an apparent local pH. Using this approach, we show that the interfacial pH on Ti_2_NT_*x*_ is both potential-dependent and time-dependent. In a nominally neutral phosphate buffer, cathodic polarization drives the interface into a substantially more alkaline regime, demonstrating that a neutral bulk pH does not ensure a neutral reaction environment. Under fixed-potential operation, this alkalization develops gradually with time, especially at more negative potentials, indicating that proton consumption can outpace buffer replenishment near the catalyst surface. Starting from a strongly acidic phosphate buffer maintains an acidic interface, but this condition risks promoting non-catalytic lattice degradation and NH_3_ release. In contrast, a mildly acidic phosphate buffer gives a more balanced response. Under cathodic polarization, the interface shifts upward toward a near-neutral pH instead of remaining strongly acidic or becoming excessively alkaline. This condition can provide a more sustained proton reservoir while avoiding the harsh acidity that destabilizes the nitride lattice. We further examined how these changes in proton availability influence NH_3_ formation from Ti_2_NT_*x*_ under cathodic polarization. Overall, this work identifies interfacial proton availability as an important parameter governing lattice-nitrogen protonation under electrochemical conditions.

## Results and Discussion

Ti_2_NT_*x*_ MNene was successfully synthesized from the Ti_2_AlN MAX phase using O_2_-assisted molten-salt fluoride etching method (O_2_-MSFE).^15,16,23,24^ The X-ray diffraction (XRD) pattern of the Ti_2_AlN precursor exhibits the sharp reflections expected for the ordered layered MAX-phase structure. Following O_2_-MSFE, these characteristic Ti_2_AlN reflections are strongly suppressed, while a broadened low-angle (002) reflection associated with Ti_2_NT_*x*_ is observed (Figure S1). The corresponding morphological transformation is evident from the SEM images (Figure S2), showing that the etched material consists of thinner, sheet-like flakes. Moreover, the Raman spectrum contains several broad vibrational features between approximately 140 and 700 cm^–1^, consistent with the Raman-active lattice vibrations previously reported for Ti_2_NT_*x*_ (Figure S3).^15,16,23^ The FTIR spectrum further indicates that the etched Ti_2_NT_*x*_ surface is chemically terminated (Figure S4). The broad absorption extending through the O–H stretching region is attributed to surface hydroxyl groups and physically adsorbed or interlayer water, whereas the lower-wavenumber features are consistent with Ti–O, Ti–OH, and Ti–F containing surface environments introduced during molten-salt etching and subsequent processing. Because these bands are broad and overlapping, they are interpreted as evidence of mixed surface terminations rather than as a quantitative determination of termination composition. Overall, these results confirm the successful synthesis of surface-terminated 2D Ti_2_NT_*x*_ MNene. Previous studies on transition-metal nitrides, particularly Mo_2_N, have shown that NH_3_ formation can arise from chemical decomposition of lattice nitrogen.^10^ We therefore first examined whether Ti_2_NT*_x_* releases NH_3_ during simple electrolyte exposure in the absence of applied potential (Figure S5). After 15 days of immersion, Ti_2_NT_*x*_ produced only a negligible amount of NH_3_ in near-neutral 0.1 M Na_2_SO_4_ electrolyte, consistent with the chemical stability of Ti_2_NT_*x*_ under neutral conditions reported in our previous study.^16^ In contrast, both 0.1 M HCl and 0.1 M KOH led to substantially higher NH_3_ accumulation, indicating that extreme pH environments can chemically activate or destabilize lattice nitrogen. XRD further revealed that the degradation pathway depends on electrolyte pH (Figure S6). In 0.1 M HCl, the Ti_2_NT_*x*_ (002) reflection was strongly suppressed, suggesting loss of layered ordering and possible structural degradation. In 0.1 M KOH, the (002) reflection was still retained, despite the large amount of NH_3_ detected. This indicates that alkaline media can promote nitrogen release without complete collapse of the layered framework. On the other hand, neutral electrolyte preserved the (002) peak, indicating that Ti_2_NT_*x*_ is comparatively stable in near-neutral electrolyte, but that both acidic and alkaline exposure can compromise lattice-nitrogen stability. Photographs of the Ti_2_NT_*x*_-coated gas-diffusion electrodes before and after immersion also revealed clear electrolyte-dependent changes in the appearance and integrity of the catalyst coating (Figure S7), consistent with the structural instability observed under strongly acidic and alkaline conditions, whereas the coating exposed to near-neutral Na_2_SO_4_ showed comparatively limited changes. These static measurements demonstrate that prolonged exposure to strongly acidic or alkaline bulk environments can promote lattice-N release through chemical pathways. However, these conditions are not equivalent to electrochemical operation, where the local environment near the Ti_2_NT_*x*_ surface can evolve dynamically as current flows. Therefore, the chemical response observed during static immersion cannot be directly used to describe the proton environment experienced by Ti_2_NT_*x*_ under cathodic polarization, indicating the necessity for direct measurement of the interfacial pH under applied potential.

The pH-dependent stability of Ti_2_NT_*x*_ highlights the urgent need to determine the local electrolyte environment experienced by the catalyst during the electrochemical polarization. Therefore, to understand how local proton availability evolves near Ti_2_NT_*x*_ and how it may regulate lattice-nitrogen protonation, we established a custom in situ ATR-FTIR configuration using a diamond ATR crystal (Figure 1a) to monitor interfacial pH using phosphate speciation as an infrared reporter. The measurement principle is shown in Figure 1a. In this configuration, the working electrode is positioned against the ATR crystal, allowing the evanescent infrared field to probe the thin electrolyte region between the catalyst film and crystal surface. Phosphate was selected as the pH reporter because its protonation state changes predictably with pH, and each acid–base form exhibits distinct vibrational fingerprints. The relevant equilibria are H_3_PO_4_/H_2_PO_4_^–^, H_2_PO_4_^–^/HPO_4_^2–^, and HPO_4_^2–^/PO_4_^3–^, with the H_2_PO_4_^–^/HPO_4_^2–^ transition being most relevant near-neutral pH. To calibrate the phosphate response, FTIR spectra were first collected from phosphate-containing electrolytes at known bulk pH values. As shown in Figure 1b, the phosphate vibrational features change systematically with pH. At low pH, bands associated with more protonated phosphate species dominate, while an increase in pH shifts the spectral response toward deprotonated phosphate species. In the near-neutral region, the relative contributions of H_2_PO_4_^–^ and HPO_4_^2–^ change strongly, providing a sensitive spectral handle for pH determination. The band assignments and peak area trends were then used to extract FTIR-derived phosphate fractions, which were compared with calculated phosphate speciation from acid–base equilibria (Figure 1c). The agreement between the calculated and FTIR-derived fractions confirms that phosphate fingerprints can be used to determine the interfacial pH within the ATR sampling region. The photograph of the custom-designed ATR-FTIR cell used for these measurements is shown in Figure S8. This custom electrochemical configuration allows simultaneous electrochemical control and spectroscopic detection while maintaining a defined working electrode position relative to the ATR crystal. Because the experiment relies on probing the near-electrode electrolyte layer, the electrode-ATR spacing is a critical aspect of the measurement. To verify that the observed phosphate response reflects the interfacial region rather than the bulk electrolyte, we performed a contact-control experiment in which the electrode was first pressed against the ATR crystal and then lifted away during subsequent cycles (Figure S9). When the electrode was pressed against the ATR crystal, the phosphate band intensities changed strongly during electrochemical cycling and the extracted pH evolved dynamically. After the electrode was lifted from the ATR surface, the phosphate bands and derived pH became nearly invariant and resembled the bulk value despite continued cycling, suggesting that the measured phosphate response is dominated by the electrolyte region adjacent to the electrode under the ATR-contact configuration.

**Figure 1:**
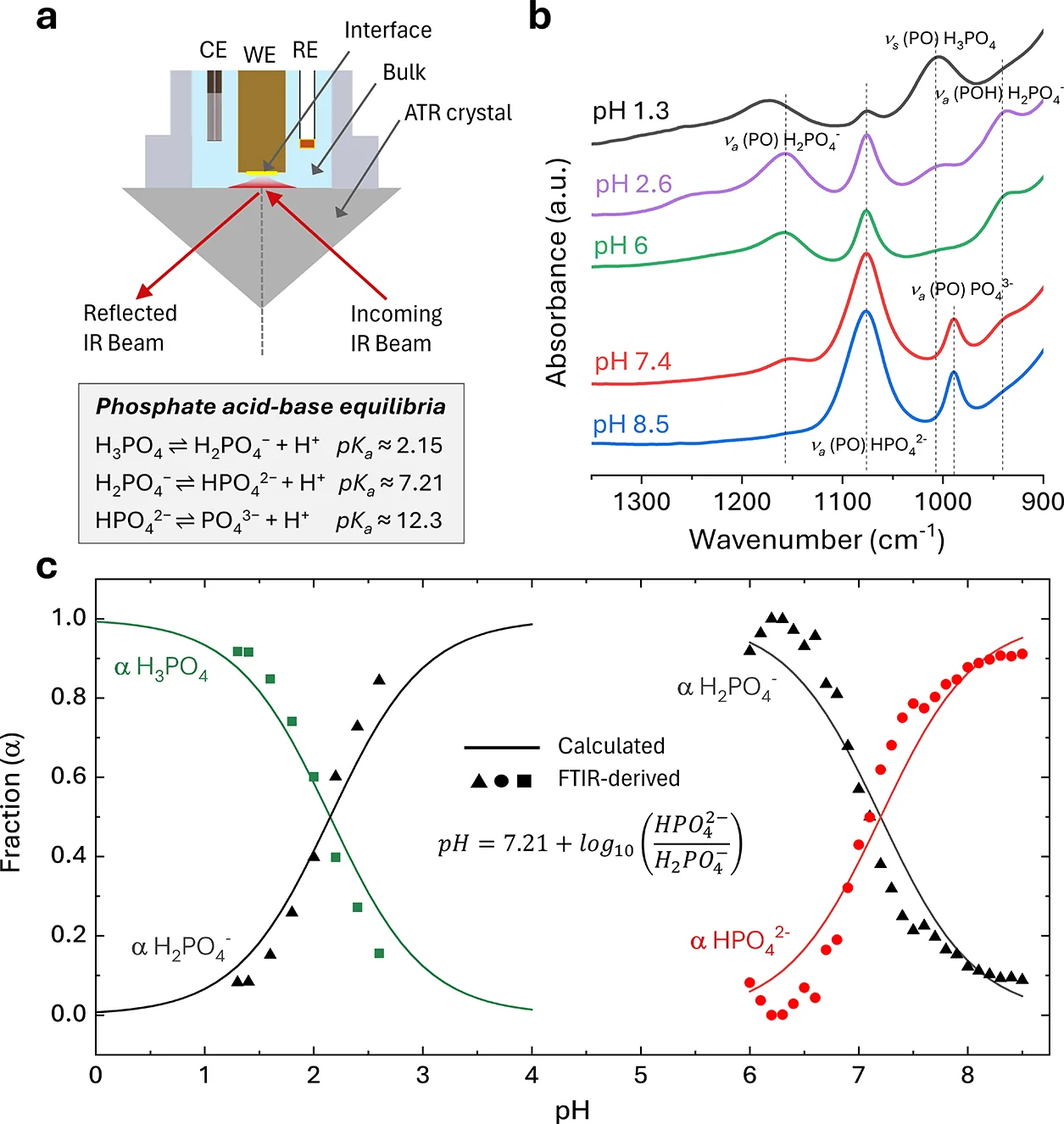
Phosphate fingerprints enable FTIR-based interfacial pH measurement. (a) Schematic of the in situ ATR-FTIR electrochemical configuration used to probe the catalyst/electrolyte interface. (b) Representative phosphate FTIR spectra collected at different pH values, showing systematic changes in vibrational features associated with H_3_PO_4_, H_2_PO_4_^–^, HPO_4_^2–^, and PO_4_^3–^ species. (c) Phosphate fractions extracted from FTIR band intensities compared with calculated equilibrium speciation. The agreement between calculated and FTIR-derived speciation validates the use of phosphate vibrational fingerprints as a pH reporter.

After establishing phosphate speciation as an FTIR-based pH reporter, we next applied this method to monitor potential-dependent interfacial pH evolution on the Ti_2_NT_*x*_ electrode. We first examined the local pH response in phosphate buffer at a pH of 7.4. Interfacial pH measurements were performed by using staircase voltammetry coupled with in situ ATR-FTIR spectroscopy. At each applied potential, the potential was held until an FTIR spectrum was collected, after which the potential was stepped to the next value. This method allows each spectrum to be associated with a defined electrode potential, enabling direct correlation among the electrochemical response, phosphate speciation, and the extracted interfacial pH.

Figure 2a shows the potential-dependent phosphate spectra collected on Ti_2_NT_*x*_ in 0.5 M phosphate buffer at pH 7.4. In the stacked spectra, the color progression follows the potential sequence, allowing the spectral evolution to be compared to the corresponding current density and calculated pH. At open circuit and positive potentials, the phosphate spectrum contains contributions from both H_2_PO_4_^–^ and HPO_4_^2–^, consistent with the near-neutral bulk pH. As the potential is swept cathodically, the phosphate bands evolve strongly, with the HPO_4_^2–^ feature becoming increasingly dominant, while the H_2_PO_4_^–^ contribution decreases, indicating a shift in phosphate speciation toward the more deprotonated form. This spectral change is the direct infrared signature of local alkalization at the Ti_2_NT_*x*_/electrolyte interface. Moreover, the appearance and growth of the more deprotonated phosphate response show that the catalyst surface does not remain at the initial bulk pH during reductive polarization. The corresponding staircase voltammogram is shown in Figure 2b. The increasing cathodic current with an increasing negative potential indicates enhanced reductive processes at the electrode, which consume protons and/or reduce water near the surface. As expected, the extracted pH profile in Figure 2c reveals a correlation to this observed current-density change. Although the bulk electrolyte was initially pH 7.4, the apparent interfacial pH increased substantially during cathodic polarization, approaching alkaline values near pH 9.5–10 at negative potentials, indicating that even in a nominally neutral phosphate buffer, the Ti_2_NT_*x*_ interface becomes significantly alkaline under cathodic polarization. This strong local alkalization indicates substantial proton depletion near the Ti_2_NT_*x*_ surface and can therefore reduce the proton availability required for lattice N protonation. Therefore, a neutral bulk electrolyte may be misleading, as the catalyst can experience a much more alkaline local environment during operation, which may alter the protonation of lattice nitrogen, suppress the forward NH_3_-forming step, or promote pH-dependent changes in the catalyst surface. The HER likely contributes importantly to this cathode-induced proton depletion. At sufficiently negative potentials, proton or water reduction associated with HER continuously consumes proton donors near the Ti_2_NT_*x*_ surface and generates OH^–^, thereby driving the interfacial electrolyte toward a higher pH. Therefore, the observed alkalization can be interpreted as a dynamic proton-depletion layer, and its magnitude reflects the balance between reductive proton consumption and replenishment from the bulk electrolyte. This effect is particularly relevant for Ti_2_NT_*x*_ because lattice-N protonation and HER compete for the same local proton reservoir.

**Figure 2:**
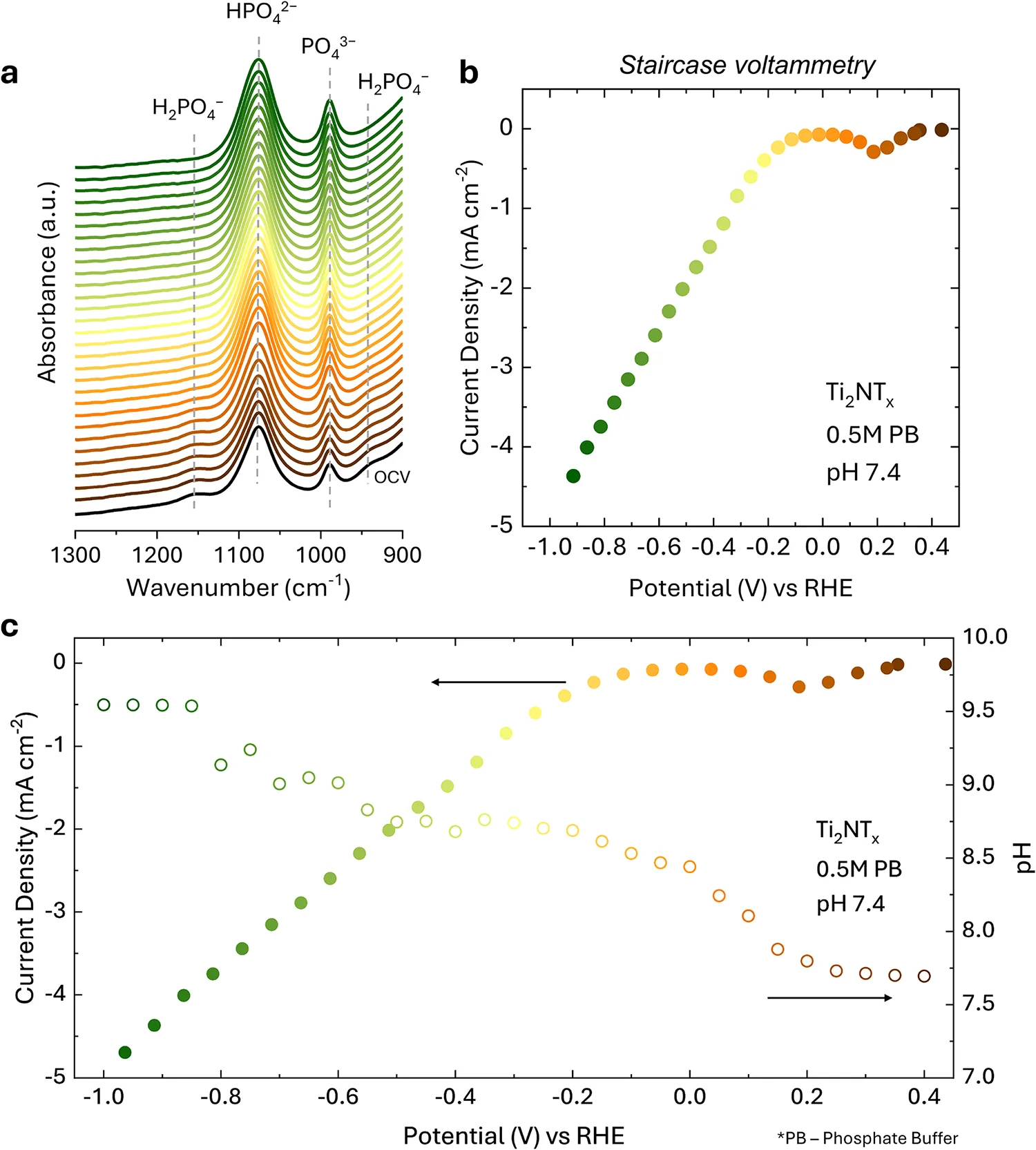
Potential-dependent interfacial pH evolution on Ti_2_NT_*x*_ in neutral phosphate buffer. (a) In situ ATR-FTIR spectra collected during staircase voltammetry on Ti_2_NT_*x*_ in 0.5 M phosphate buffer at pH 7.4. (b) Staircase voltammogram showing the corresponding potential-dependent current density. (c) Overlay of current density and FTIR-derived apparent interfacial pH as a function of potential.

To further confirm that the observed cathodic alkalization is not caused by the high phosphate concentration, an additional control experiment was performed using 0.1 M KCl mixed with only 0.05 M phosphate buffer at an initial pH of 7.4 (Figure S10). Sulfate electrolyte was not used here because of overlapping vibrational modes with the phosphate region used for pH analysis. Figure S10a shows that, even at the lower phosphate concentration, the phosphate bands still shifted toward more deprotonated species (PO_4_^3–^) during cathodic polarization. This is consistent with the spectral trend observed in the 0.05 M phosphate calibration spectra as the bulk pH was increased (Figure S11). However, because the H_2_PO_4_^–^ band was weak at this lower phosphate concentration, reliable quantitative pH extraction was not possible. Therefore, this experiment was used only as a qualitative control, showing that cathodic alkalization is still observed when phosphate is present at a much lower concentration in a neutral supporting electrolyte.

To determine whether increased proton availability could moderate this cathodic alkalization, the same experiment was performed in 0.5 M phosphate buffer adjusted to pH 4.7 (Figure 3). The spectra again show clear potential-dependent changes in phosphate speciation, but the response differs from that of the pH 7.4 case (Figure 3a). At pH 4.7, the initial phosphate environment is more protonated, with stronger H_2_PO_4_^–^ character, which, upon cathodic polarization, shifted toward increased HPO_4_^2–^ contribution, indicating that the interface still becomes more alkaline as reductive current flows. The corresponding staircase voltammogram shows an increase in cathodic current density with increasing negative potential (Figure 3b), consistent with increasing proton consumption and/or water reduction at the electrode surface. The interfacial pH extracted from the phosphate band ratios rises well above the initial bulk value, reaching near-neutral to mildly alkaline values at negative potentials (Figure 3c). Therefore, mild acidification increases the proton reservoir but does not fully prevent the cathode-side alkalization. Proton consumption at the Ti_2_NT_*x*_ interface can still outpace proton transport and buffer replenishment under sufficiently reducing conditions.

**Figure 3:**
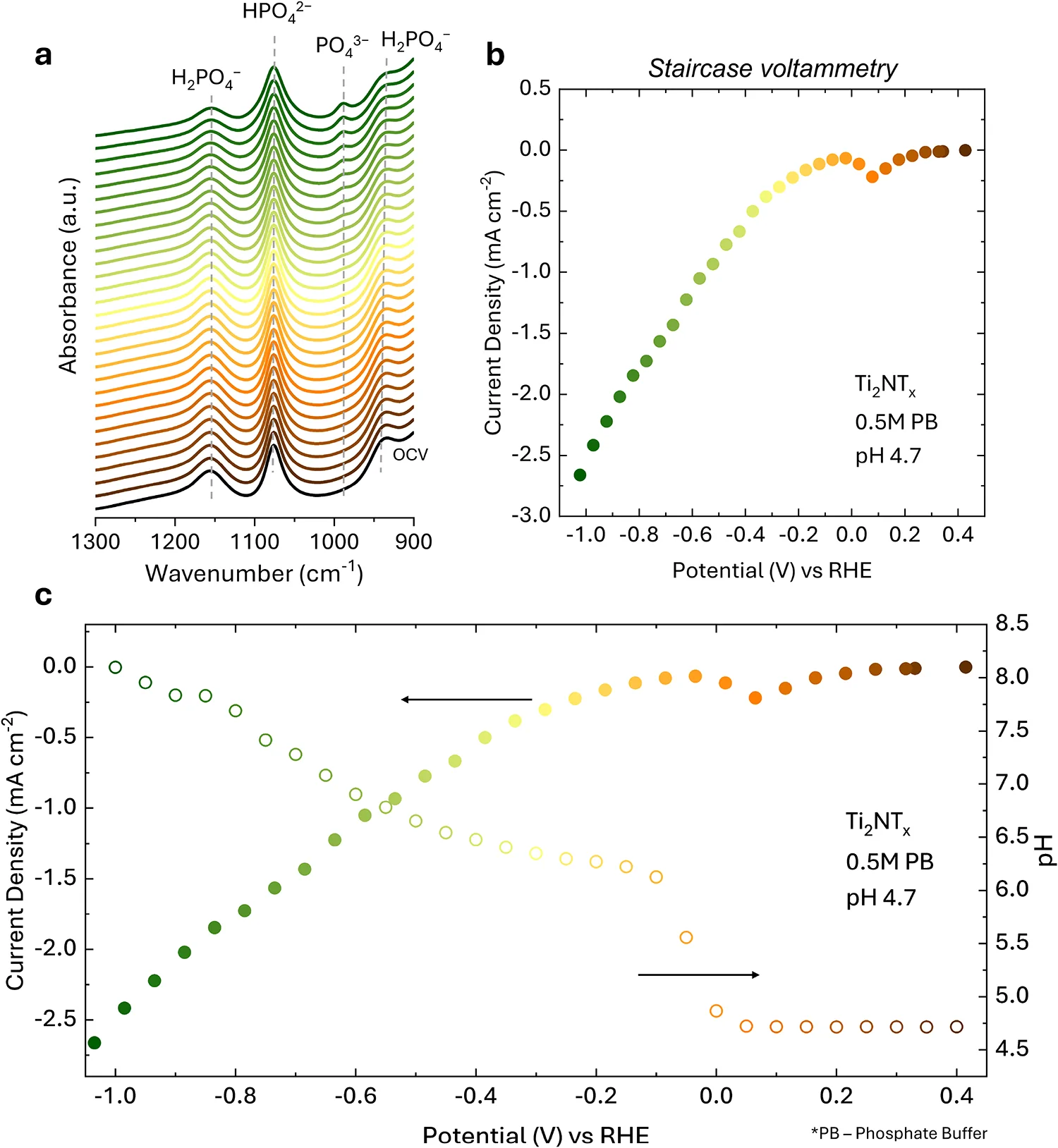
Potential-dependent interfacial pH evolution on Ti_2_NT_*x*_ in mildly acidic phosphate buffer. (a) In situ ATR-FTIR spectra collected during staircase voltammetry on Ti_2_NT_*x*_ in 0.5 M phosphate buffer at pH 4.7. (b) Staircase voltammogram showing the corresponding potential-dependent current density. (c) Overlay of current density and FTIR-derived apparent interfacial pH as a function of potential.

Finally, we examined a strongly acidic phosphate electrolyte at pH 1.3 (Figure 4). In this case, the phosphate spectra show substantially smaller potential-dependent changes than those observed at pH 7.4 and 4.7, with the protonated phosphate species dominating throughout polarization (Figure 4a). The staircase voltammogram nevertheless shows a marked increase in cathodic current with increasing negative potentials, particularly below approximately −0.6 V vs RHE (Figure 4b). Despite this increase in reductive current, the extracted interfacial pH changes only slightly and remains strongly acidic across the potential range (Figure 4c). This indicates that the high proton availability in a strongly acidic electrolyte can rapidly neutralize cathodically generated alkalinity, maintaining a more constant interfacial pH. In other words, the proton supply is sufficiently large that the local pH deviations are minimized. However, maintaining high proton availability is not necessarily desirable because the static immersion experiments show that strongly acidic conditions can directly promote chemical protonation and release of lattice nitrogen as NH_3_. More importantly, these potential-dependent measurements show that the interfacial pH on Ti_2_NT_*x*_ is controlled by a balance between cathodic proton consumption and proton replenishment from the electrolyte.

**Figure 4:**
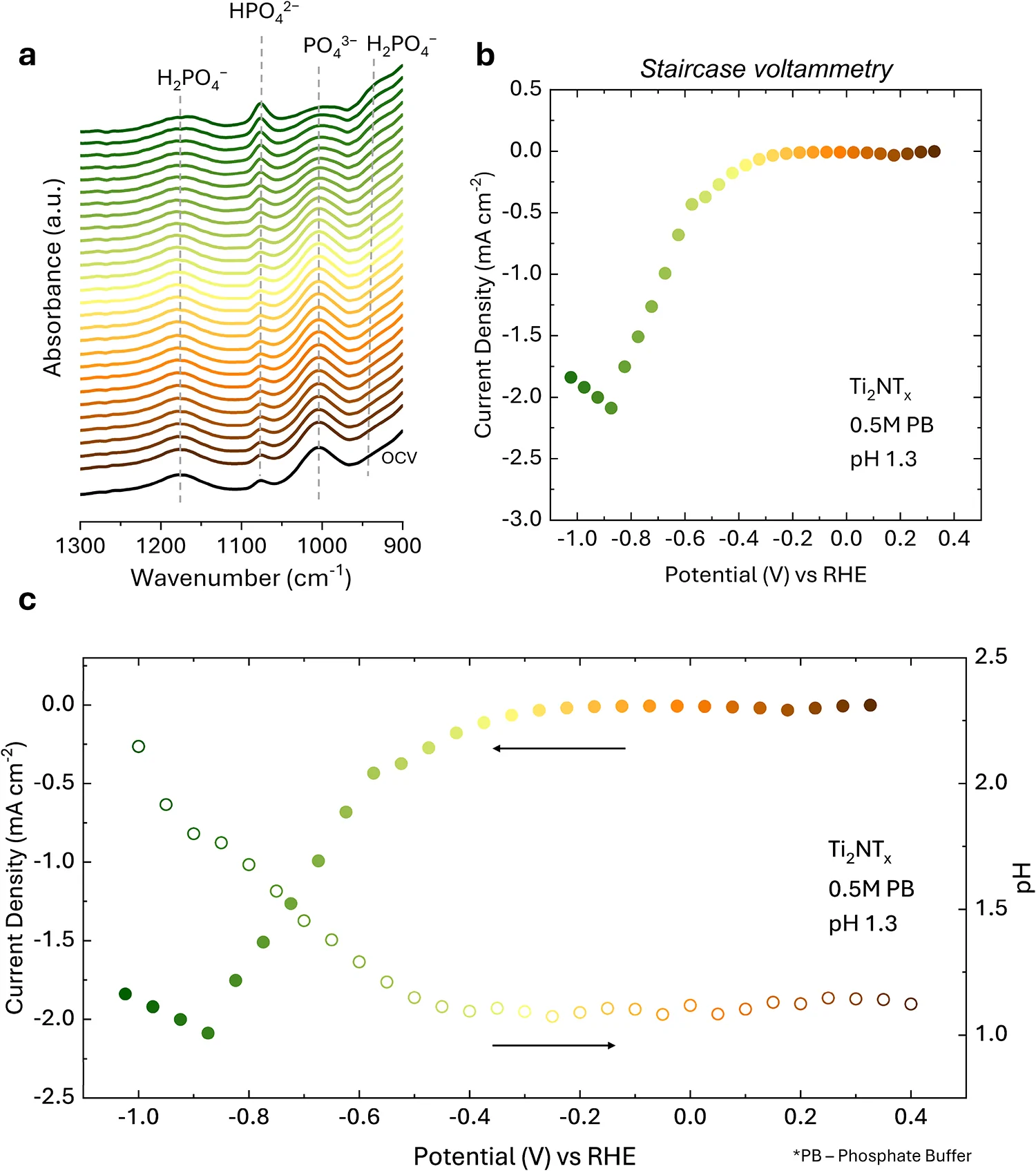
Potential-dependent interfacial pH evolution on Ti_2_NT_*x*_ in strongly acidic phosphate buffer. (a) In situ ATR-FTIR spectra collected during staircase voltammetry on Ti_2_NT_*x*_ in 0.5 M phosphate buffer at pH 1.3. (b) Staircase voltammogram showing the corresponding potential-dependent current density. (c) Overlay of current density and FTIR-derived apparent interfacial pH as a function of potential.

To further distinguish between instantaneous spectral changes and time-dependent electrolyte restructuring, we monitored the temporal evolution of the interfacial pH at fixed applied potentials in a pH 7.4 phosphate buffer (Figure 5). At each potential, the current density decayed rapidly at early times and then approached a quasi-steady value, whereas the FTIR-derived pH evolved more gradually. This difference in time scale indicates that the interfacial pH is determined by the applied potential as well as the balance between cathodic alkalinity generation and electrolyte-mediated neutralization. At potentials near 0 to −0.2 V vs RHE, the interfacial pH remained close to the starting bulk value, showing only minor changes over time. However, at more negative potentials, the local pH increased progressively, with the largest rise observed at −0.5 V vs RHE. Under these conditions, the interfacial pH continued to increase over the measurement window, reaching values above pH 8.5 despite the bulk electrolyte initially being pH 7.4. Such a delayed pH response compared with the rapid current decay suggests that OH^‑^ accumulation/proton depletion develops over time as reductive chemistry proceeds, while neutralization by the phosphate buffer is not instantaneous. This temporal behavior is consistent with the staircase voltammetry measurements, as the cathodic polarization drives the Ti_2_NT_*x*_ interface to be alkaline, and the magnitude of alkalization increases with the rate of reductive current.

**Figure 5:**
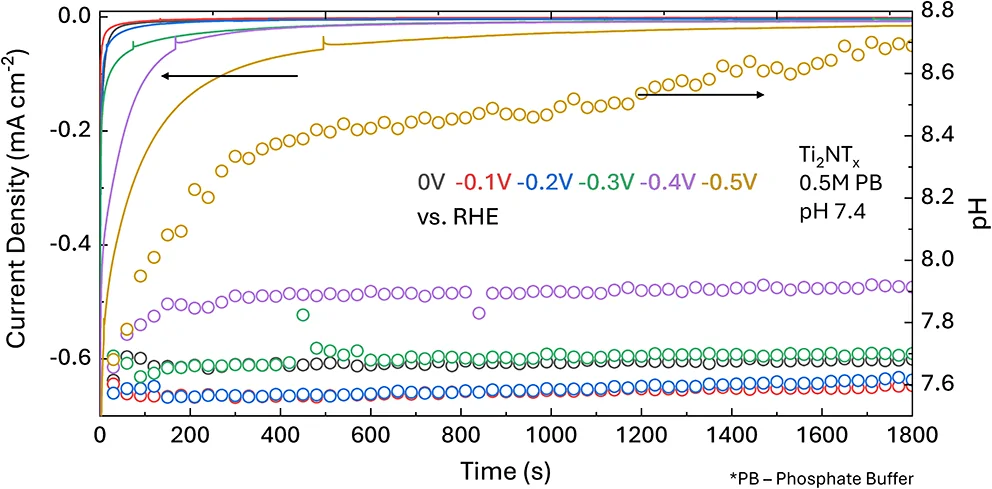
Time-dependent current density and interfacial pH measured on Ti_2_NT_*x*_ during potentiostatic polarization in 0.5 M phosphate buffer at an initial pH of 7.4. The electrode was held at 0, −0.1, −0.2, −0.3, −0.4, and −0.5 V vs RHE for 1800 s. Solid lines show the corresponding current-density profile and the open circles show the interfacial pH extracted from phosphate speciation.

The competition between alkalinity generation and electrolyte neutralization was further visualized using an H-cell colorimetric pH experiment (Figure S12). Here, RuO_2_ electrodes were used in both compartments, and electrolysis was performed at a fixed current density of 10 mA cm^–2^, and the initial H_2_SO_4_/Na_2_SO_4_ ratio was varied. Because the current density was held constant, the rate of cathodic alkalinity generation was fixed, allowing for the effect of proton supply and neutralization capacity to be compared directly. In electrolytes with a lower acid content, the catholyte rapidly shifted to alkaline colors, while the anolyte remained comparatively acidic. Increasing the H_2_SO_4_ fraction delayed and suppressed the catholyte alkalization, indicating that a larger proton reservoir can partially compensate for cathodic OH^‑^ generation. However, the persistence of visible pH gradients under several compositions shows that proton supply does not instantaneously eliminate local alkalization when a reductive current is continuously imposed. This further supports the interpretation that the interfacial pH is governed by the balance between current-driven alkalinity production and electrolyte-mediated neutralization.

To examine how the initial proton availability influences NH_3_ formation from Ti_2_NT_*x*_ under cathodic polarization, electrolysis was performed at −0.5 V vs RHE for 4 h in 0.5 M Na_2_SO_4_ at initial pH values of 7.2 and 4.8 under Ar and N_2_ atmospheres (Figure 6). The result shows that NH_3_ was detected under both gas environments, and lowering the initial electrolyte pH increased NH_3_ formation under both Ar and N_2_, indicating that the response is strongly influenced by proton availability. In the context of the interfacial pH measurements, these results suggest that strong cathodic alkalization can reduce the proton availability required for lattice N protonation, whereas mild acidification can partially compensate for this proton depletion. Additional control experiments were performed to evaluate the contribution of background NH_3_ and passive lattice-N release (Figure S13). The blank electrolyte and GDL-only controls showed substantially lower NH_3_ signals than Ti_2_NT_*x*_ polarized at −0.5 V vs RHE. Also, Ti_2_NT_*x*_ maintained at an open-circuit potential produced a much smaller signal than under cathodic polarization, indicating that the NH_3_ formation observed under applied potential is primarily associated with electrochemically driven protonation of Ti_2_NT_*x*_. Importantly, these controls do not establish the nitrogen source of the electrochemically generated NH_3_, and the differences between Ar and N_2_ are therefore not interpreted mechanistically. To further investigate whether the pH-dependent NH_3_ trend was specific to the Na_2_SO_4_ electrolyte, electrolysis was performed directly in 0.5 M phosphate buffer under the same applied potential and electrolysis time (Figure S14). Similar to the 0.5 M Na_2_SO_4_ measurements, lowering the initial pH from 7.4 to 4.8 increased the level of NH_3_ formation under both Ar and N_2_. Although the absolute NH_3_ values differ between the two electrolyte systems, the same qualitative dependence on the initial proton availability is retained. This also suggests that the observed increase in NH_3_ formation at a lower initial pH is not unique to the sulfate electrolyte. Overall, pH tuning must be interpreted as a balance between facilitating catalytic lattice-N protonation and avoiding excessive non-catalytic lattice-nitrogen loss.

**Figure 6:**
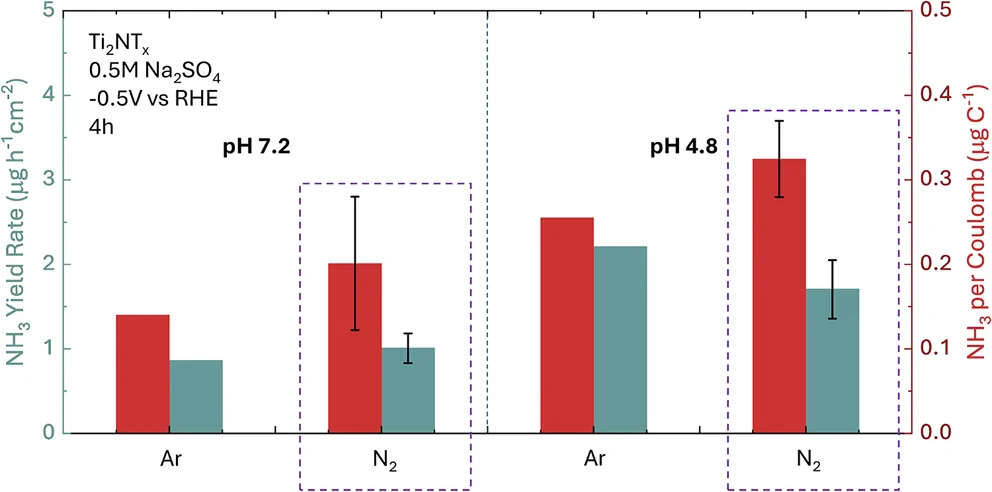
Effect of initial electrolyte pH on NH_3_ formation from Ti_2_NT_*x*_ during cathodic electrolysis under Ar and N_2_. Electrolysis was performed for 4 h at −0.5 V vs RHE in 0.5 M Na_2_SO_4_ adjusted to initial pH values of 7.2 and 4.8. NH_3_ formation rate and NH_3_ produced per coulomb are shown for each condition.

## Summary

Here, we show that cathodic alkalization is an intrinsic consequence of reductive operation in aqueous electrolytes, as current flows, proton consumption, and water reduction generate a local alkaline environment near the Ti_2_NT_*x*_ surface. The extent of this pH increase is determined by the balance between the rate of alkalinity generation and the rate of neutralization by proton donors in the electrolyte. At pH 7.4, the neutral bulk electrolyte does not supply protons rapidly enough to prevent the interface from becoming strongly alkaline. At pH 4.7, increased proton availability partially moderates the pH rise, but cathodic alkalization still occurs when the reductive current is sufficiently high. At pH 1.3, the proton supply is high enough to rapidly neutralize the alkalinity generated at the cathode, resulting in only a small interfacial pH change. These observations suggest that the electrolyte design should not simply maximize acidity or proton availability. Instead, the goal should be to maintain a near-neutral interfacial environment where proton delivery is sufficient to support lattice-nitrogen protonation but not so acidic or alkaline that the catalyst becomes chemically destabilized. Achieving this window requires coupling the initial electrolyte pH with the applied current density so that proton delivery to the lattice nitrogen is maintained without pushing the nitride lattice into strongly acidic or alkaline degradation regimes. Overall, these results show that bulk pH does not describe the environment experienced by lattice nitrogen during electrochemical operation and identify interfacial proton availability as a key parameter governing lattice-N reactivity under cathodic polarization.

## Supplementary Material



## Notes

Experimental methods; Ti_2_NT_*x*_ synthesis and characterization; static electrolyte-immersion and structural-stability measurements; ATR-FTIR cell validation and phosphate calibration; low-phosphate supporting-electrolyte control; H-cell pH visualization; NH_3_ blank, GDL, and open-circuit controls; and phosphate-buffer NH_3_ electrolysis measurements (PDF)
