# Supplementary material for: Interfacial pH Dynamics in Lattice-Nitrogen Protonation to Ammonia
Source: J Am Chem Soc. 2026 Oct 7;148(41):44739–47. doi: 10.1021/jacs.6c14168 (PMC13693450; doi:10.1021/jacs.6c14168)
Supplement: Supplementary file 1 [file ja6c14168_si_001.pdf]

Supporting Information

Interfacial pH Dynamics in Lattice-Nitrogen Protonation to Ammonia

David Kumar Yesudoss[1], Joshua Ferguson[1], Anabela Meier[2], Abdoulaye Djire[1,3*]

[1]Artie McFerrin Department of Chemical Engineering, Texas A&M University, College Station, TX 77843, USA

[2]Department of Chemistry, Texas A&M University, College Station, TX 77843, USA

[3]Department of Materials Science and Engineering, Texas A&M University, College Station, TX 77843, USA

Corresponding Authors: adjire@tamu.edu

Experimental Methods

Ti_2NT_x MNene Synthesis

Ti_2AlN Synthesis

The Ti_2AlN MAX phase was synthesized by grinding powders of Ti (Sigma-Aldrich, 99.7%, 100 mesh) and AlN (Sigma-Aldrich, 99%, 3 µm) in a 2:1 molar ratio in an agate mortar for 10 min. The mix was then sintered in a tube furnace (CM Furnace Inc. 1730-20 HT) at 1600 ºC for 1 h at a ramp rate of 10 ºC min^{-1} under constant Ar flow (Airgas, Ultra High Purity). The resulting Ti_2AlN pellet was ground in an agate mortar in preparation for etching.

Ti_2NT_x Synthesis

Ti_2NT_x MNene was synthesized by removing the aluminum (Al) layer from Ti_2AlN MAX phase using oxygen-assisted molten salt etching. The process began by thoroughly mixing 0.25 g of Ti_2AlN MAX phase with 0.25 g of a molten salt mixture (59 wt% KF, 29 wt% LiF, and 12 wt% NaF) using a pestle and mortar. The mixture was transferred to an alumina crucible and heated in a tube furnace to 550ºC at a rate of 10ºC/min under a flow of 360 SCCM argon (Ar). After reaching

550ºC, the temperature was maintained for 5 h. Halfway through the process, the argon flow was stopped, and the furnace inlet and outlet were sealed. The furnace was then cooled to 450ºC, held at this temperature for 1 h, and subsequently allowed to cool to room temperature. The etched material was extracted for further acid treatment. To remove the etched aluminum salts formed during the molten salt treatment, the etched Ti_2AlN material was dispersed in 20 mL of 4M formic acid and stirred at 600 rpm for 1 h. The suspension was vacuum filtered through a 0.4 μm polycarbonate membrane (Whatman Nucleopore), and any remaining residual salts and formic acid were removed by washing the filter membrane with 1 L of deionized (DI) water. After filtration, the membrane was dried overnight in a vacuum oven at 50 ºC. The dried material, referred to as multilayered Ti_2NT_x, was scraped from the membrane and set aside for delamination.

Delamination

To produce delaminated Ti_2NT_x MNene, the multilayered Ti_2NT_x was dispersed in 10 mL of DI water solution and stirred at 600 rpm for 4 h. The suspension was then centrifuge-washed at 3500 rpm with DI water until a neutral pH was reached. After this, the dispersion was sonicated for 1 h and allowed to rest for another hour to let any unetched MAX phase settle. The supernatant was carefully pipetted out and vacuum filtered to collect delaminated Ti_2NT_x powders.

Electrode Preparation

The working electrode was prepared by drop casting catalyst ink onto 3 mm diameter glassy carbon electrode. To prepare catalyst ink, 2 mg of MNene was mixed into 8 μL of 5 wt% Nafion in ethanol and 200 μL of ethanol, then sonicated for 30 min. 5 μL of this solution was then dropped onto the GC electrode and the electrode dried at 50 °C under vacuum for 30 min.

In situ ATR-FTIR spectroelectrochemistry

In situ attenuated total reflection Fourier transform infrared (ATR-FTIR) spectroelectrochemical measurements were performed using a Bruker INVENIO-R spectrometer equipped with a liquid nitrogen-cooled mercury cadmium telluride (MCT) detector and a diamond ATR stage. The

electrochemical cell was positioned directly on the diamond ATR crystal, which served as the infrared optical interface. In this configuration, the evanescent infrared field generated at the diamond/electrolyte interface probes the thin electrolyte region adjacent to the working electrode, enabling detection of potential-dependent changes in interfacial phosphate speciation. A custom spectroelectrochemical cell was used for all measurements (**Figure S2**). A catalyst coated glassy carbon rod was used as the working electrode. Ag/AgCl was used as the reference electrode, and a graphite rod was used as the counter electrode. The working electrode was presoaked in the electrolyte before measurements to ensure complete wetting and reproducible electrode/electrolyte contact.

Phosphate buffer electrolytes were prepared using ultrapure water (18.2 MΩ.cm) and analytical-grade reagents, and the pH was adjusted to the desired value before each experiment. Electrochemical control was performed using a BioLogic SP-300 potentiostat. All potentials were converted to the reversible hydrogen electrode (RHE) scale unless otherwise noted. Potential-dependent interfacial pH measurements were performed using staircase voltammetry. At each applied potential, the potential was held while an ATR-FTIR spectrum was collected, after which the potential was stepped to the next value. This allowed each spectrum to be correlated with a defined applied potential. For temporal measurements, the electrode was held at a fixed potential while spectra were collected continuously to monitor time-dependent phosphate speciation and apparent interfacial pH. The relative areas of pH-sensitive phosphate vibrational bands were used to extract interfacial pH values.

NH_3 Quantification

NH_3 concentration was estimated via UV-Vis spectrometry using the Berthelot test. 2 mL analyte, 1.25 mL oxidizing solution (0.625 M sodium hydroxide containing 0.36 M salicylic acid and 0.17 M sodium citrate), 0.15 mL catalyst solution (1 wt% sodium nitroferricyanide in water), and 0.075 mL oxidizing solution (4 wt% Cl of sodium hypochlorite in water) were mixed, and incubated in the dark for 2 h at room temperature. The total NH_3 formed in the solution was calculated using the following equation

$$Total\ NH_3(\mu g) = C_{NH_3} \times V$$

where C_{NH_3} is the measured NH_3 concentration and V is the volume of electrolyte in the system (in mL).

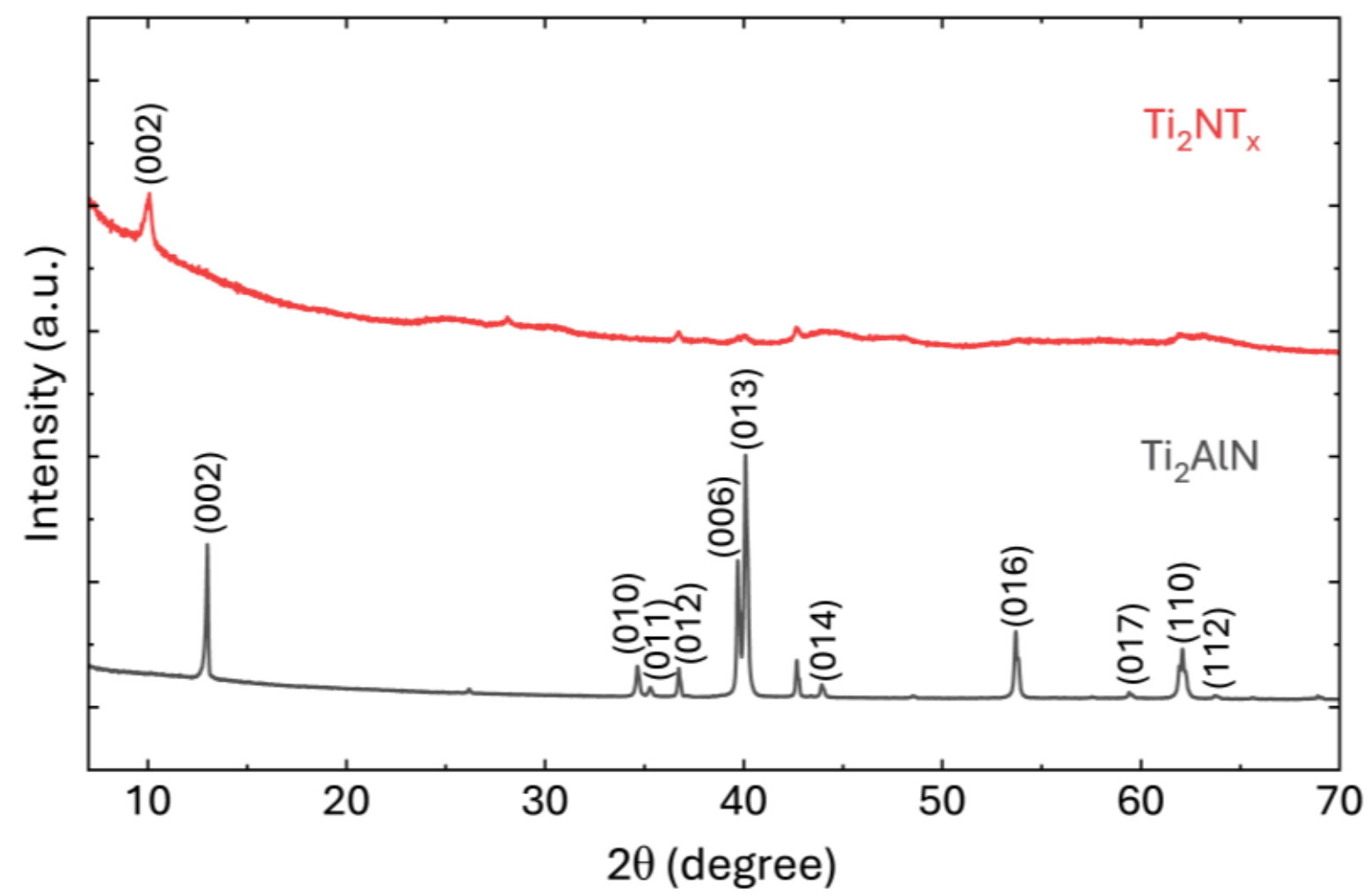

Figure S1. XRD pattern of the Ti_2AlN MAX phase and Ti_2NT_x MNene.

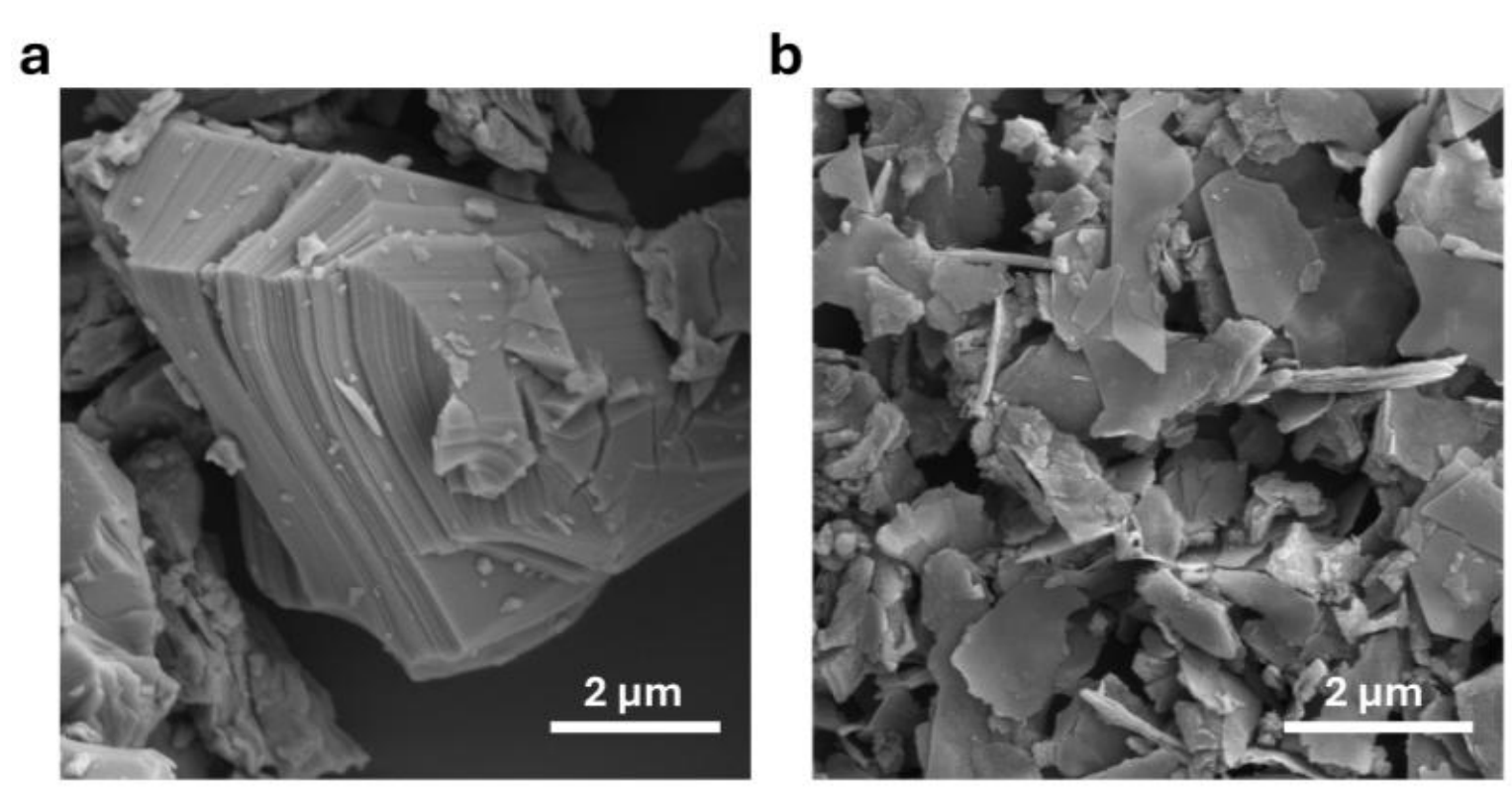

Figure S2. SEM imaging of (a) Ti_2AlN MAX phase and (b) delaminated Ti_2NT_X MNene.

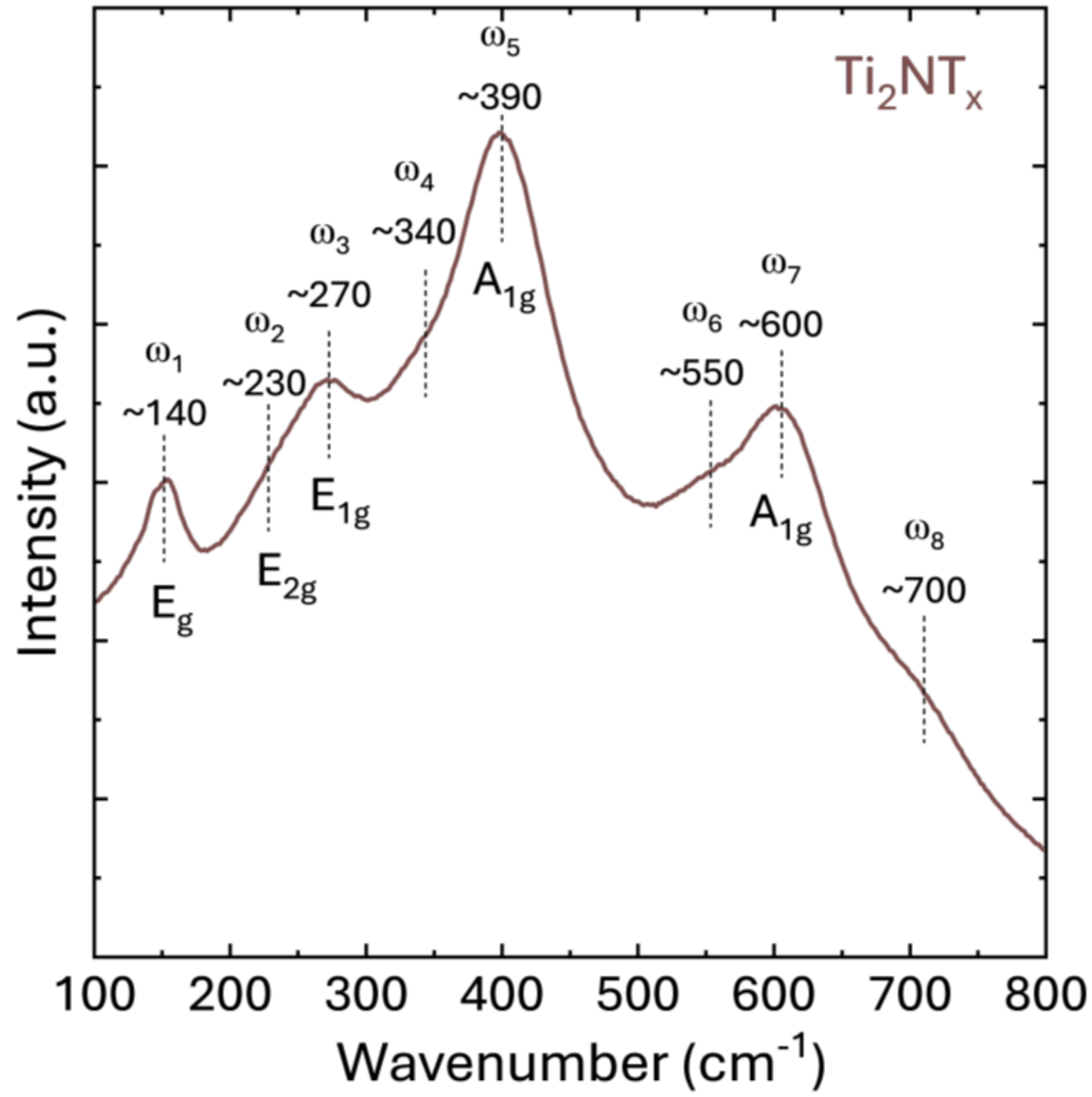

Figure S3. Raman spectra of delaminated Ti_2NT_X MNene. Raman spectroscopy was gathered using 532 nm laser at 10 % power at a 1 s exposure time.

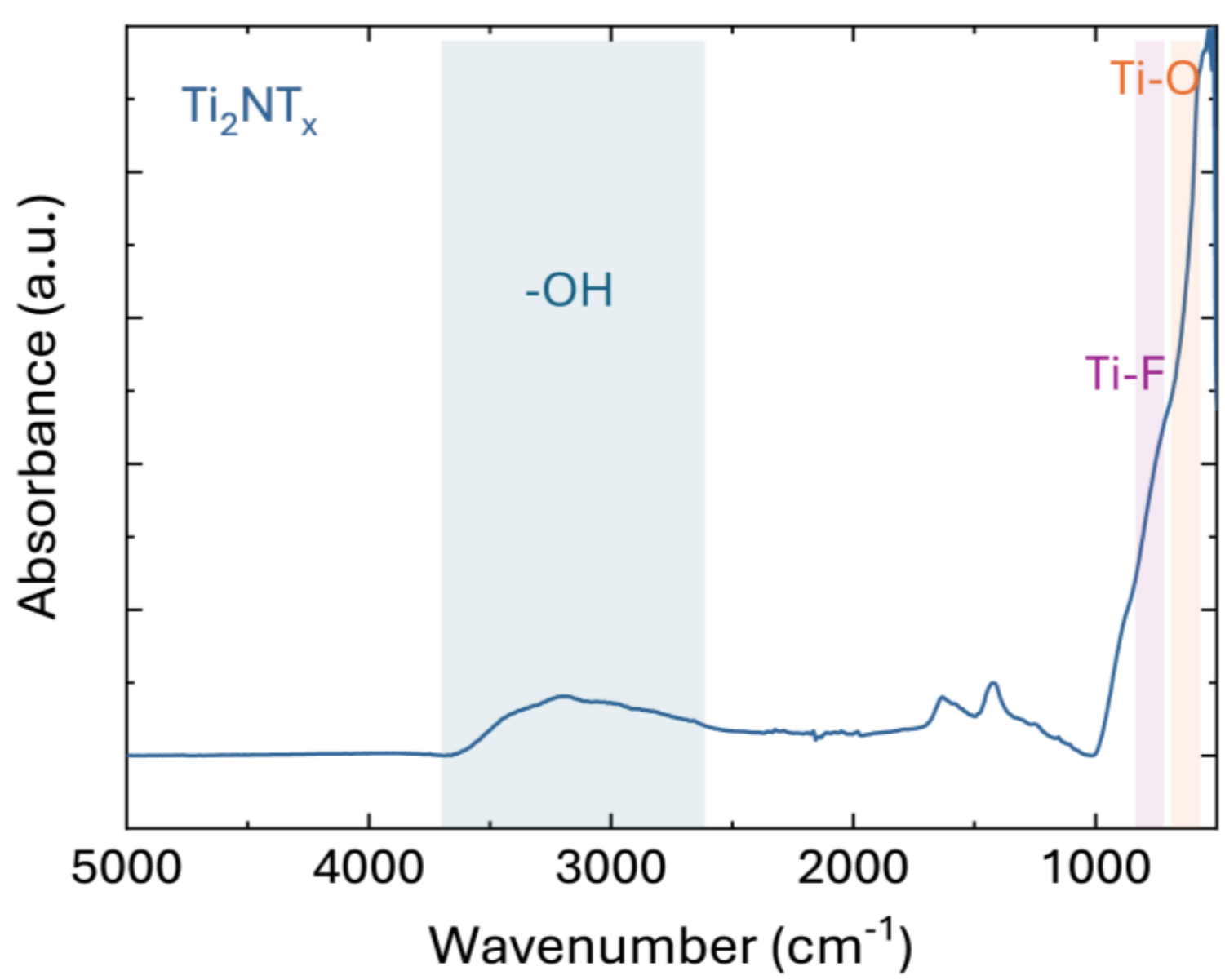

Figure S4. FTIR spectra of delaminated Ti_2NT_X MNene.

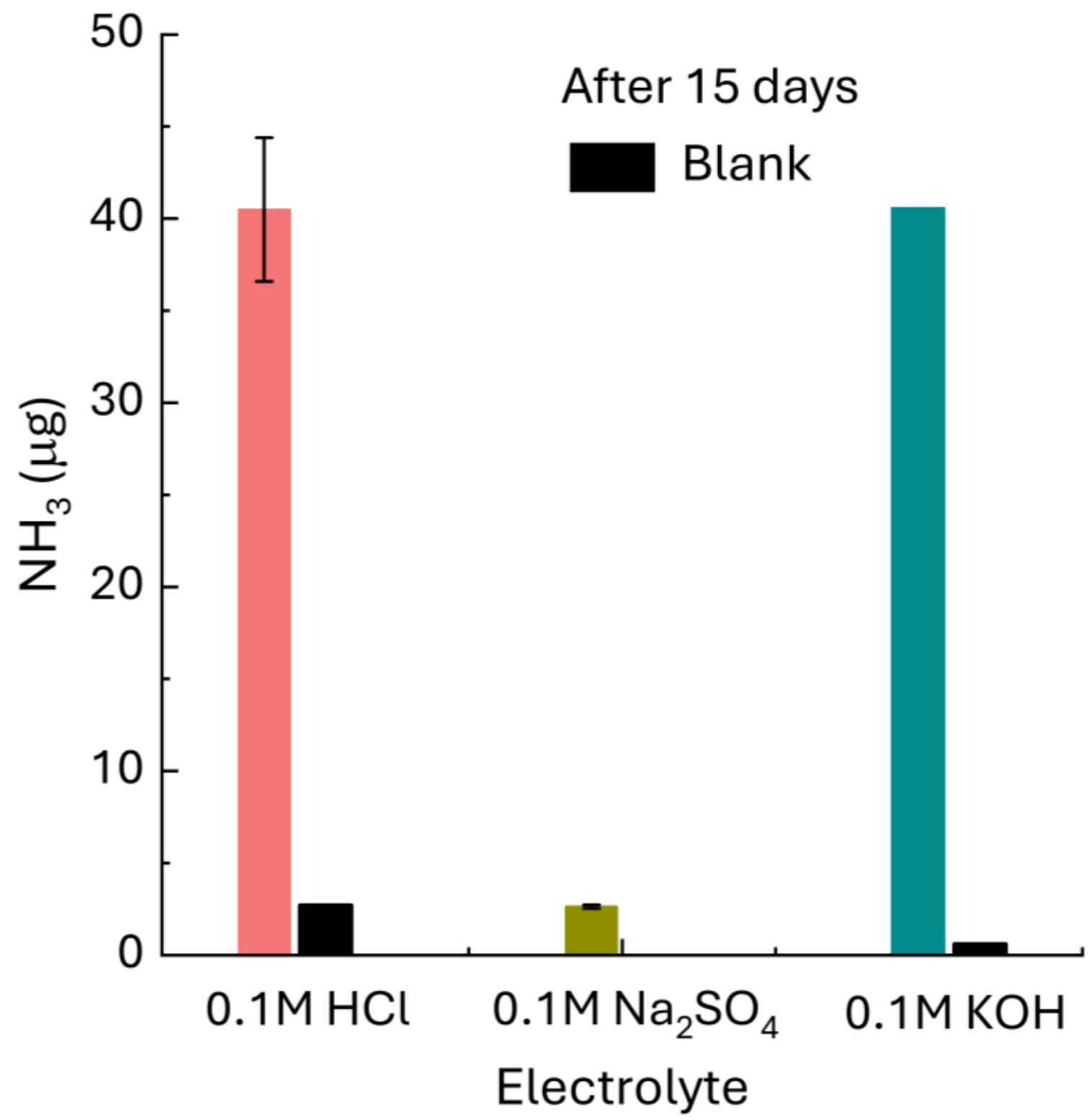

Figure S5. NH_3 detected after static immersion of Ti_2NT_x coated in gas diffusion electrode (GDE) in 0.1 M HCl, 0.1 M Na_2SO_4, and 0.1 M KOH for 15 days. Electrolyte blanks without Ti_2NT_x were measured under identical conditions using GDE only.

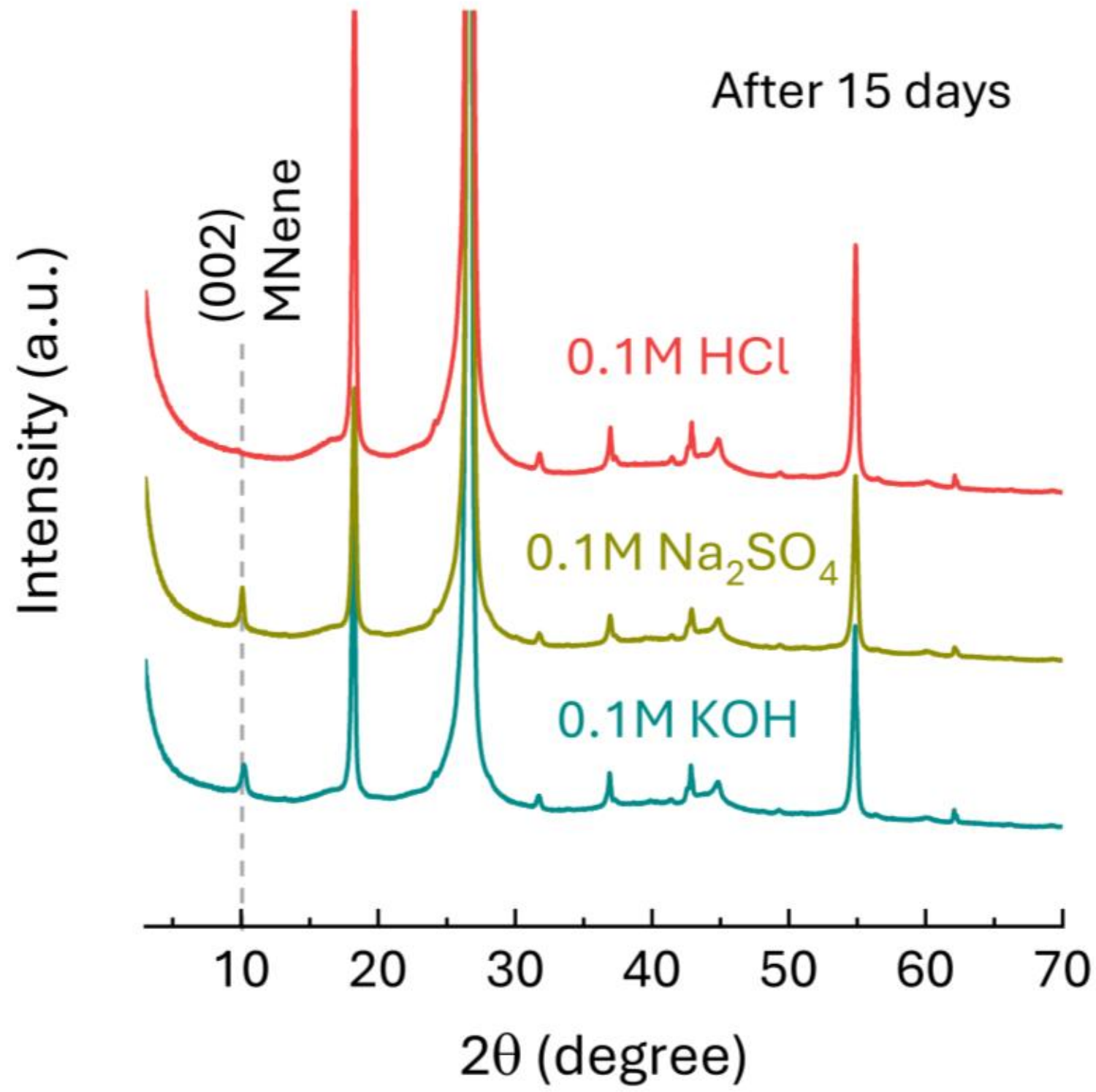

Figure S6. XRD patterns of Ti_2NT_x after 15 days of electrolyte exposure.

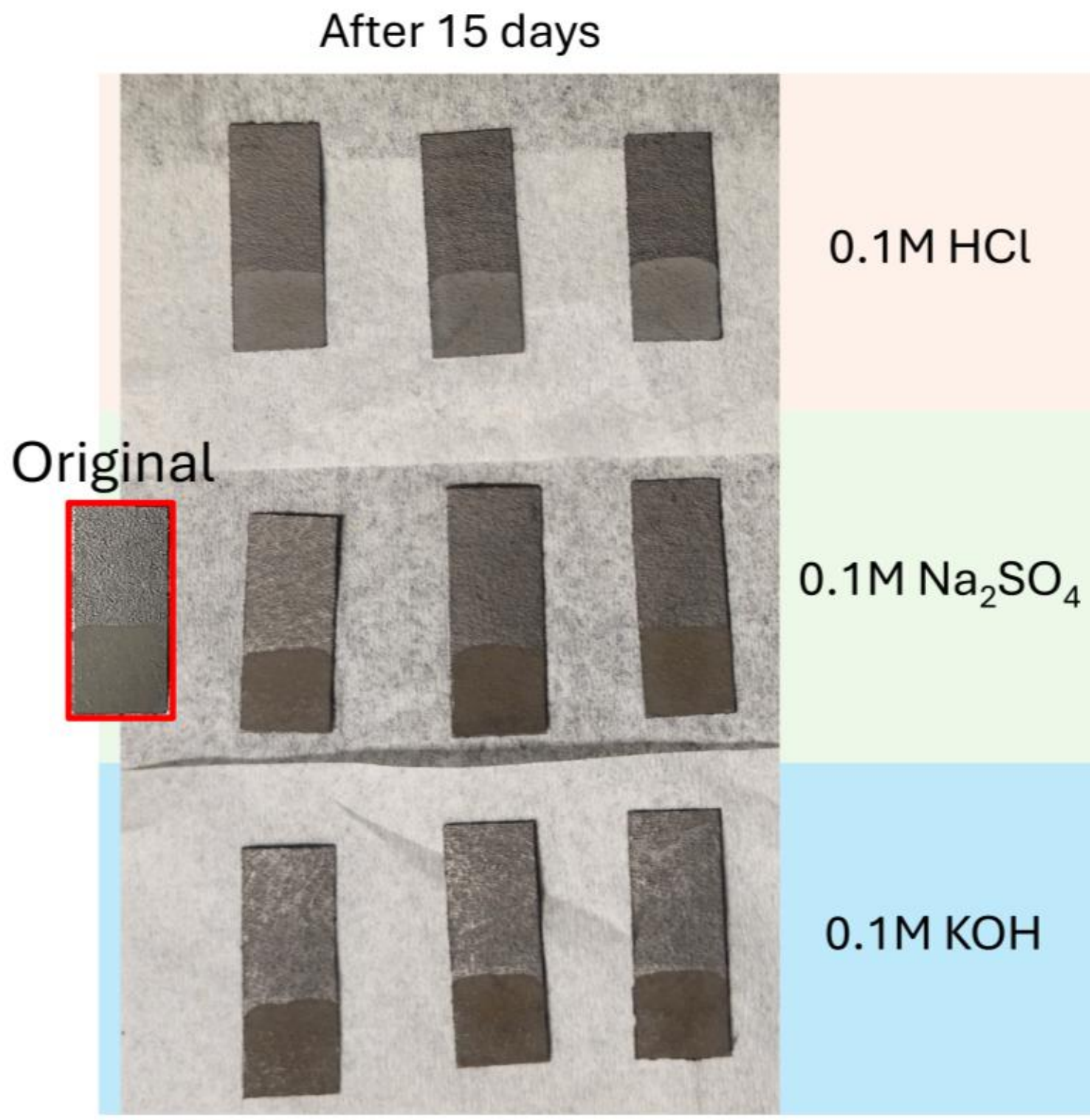

Figure S7. Photographs of Ti_2NT_x coated GDE before and after immersion in the corresponding electrolytes, showing visible electrolyte-dependent changes after prolonged exposure.

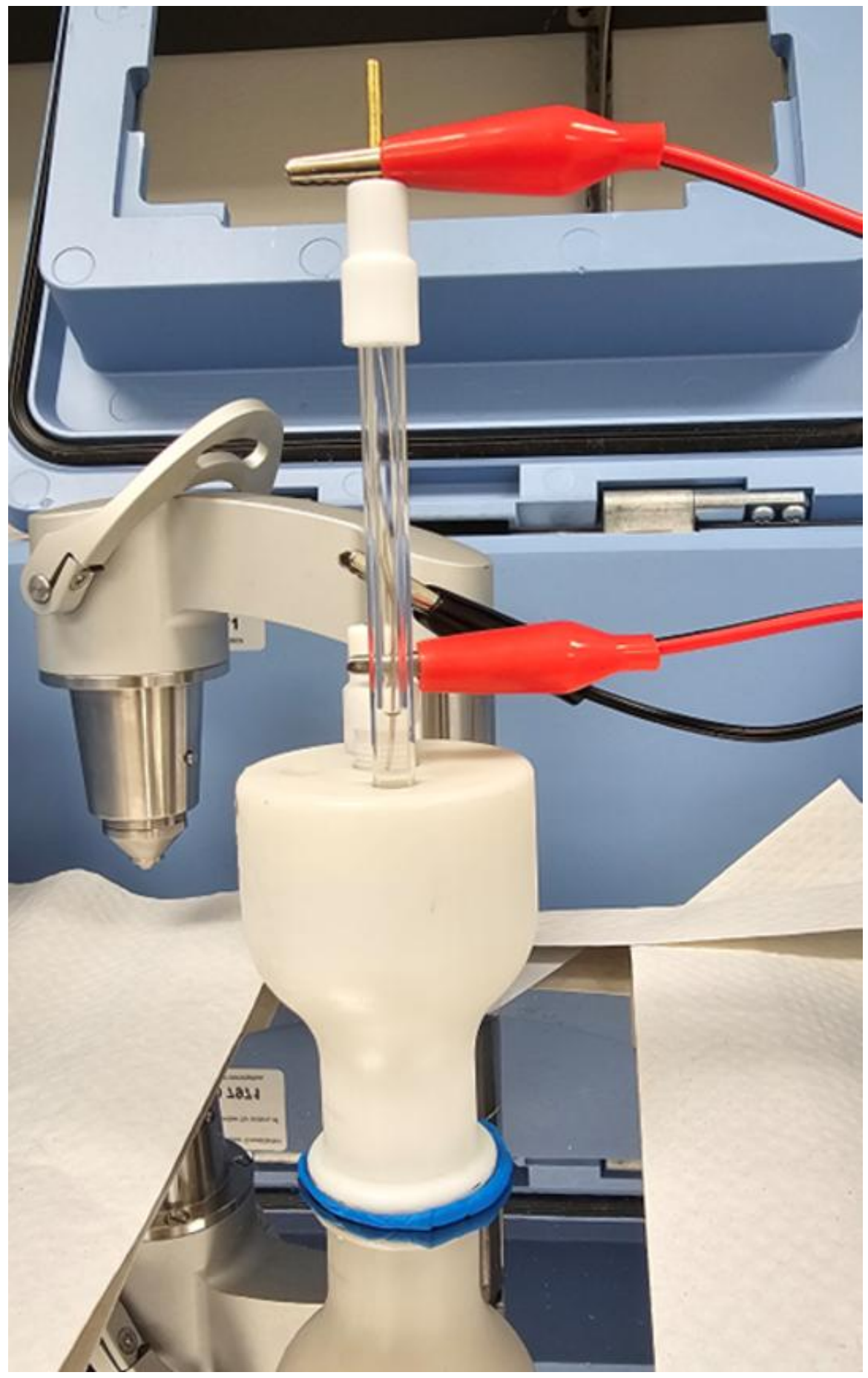

Figure S8. Photograph of the in-situ ATR-FTIR electrochemical cell.

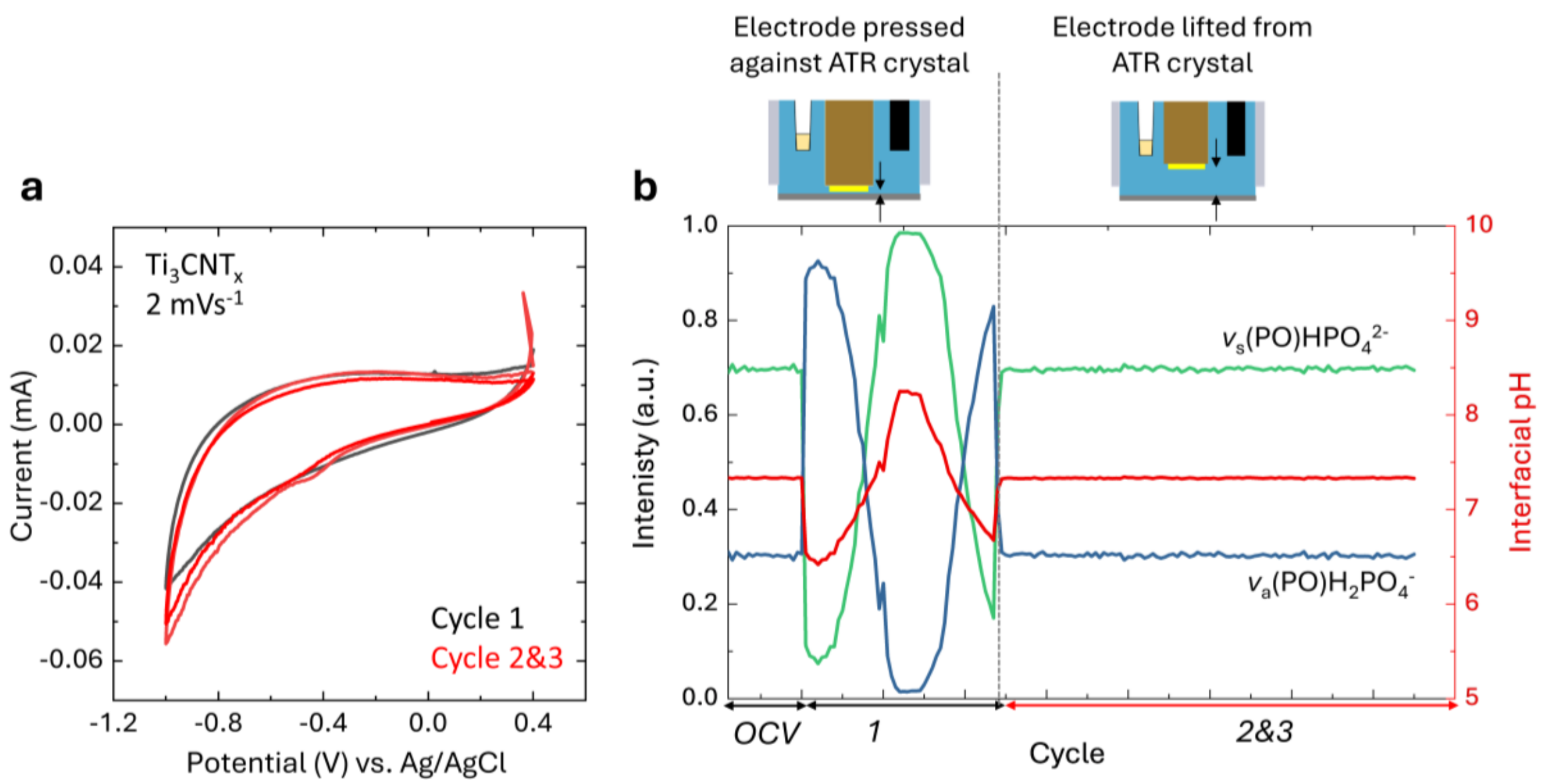

Figure S9. ATR-contact-dependent phosphate response confirms interfacial pH sensitivity. (a) Cyclic voltammograms (CV) collected during the contact-control experiment using Ti_3CNT_x as working electrode. (b) Evolution of phosphate band intensities and FTIR-derived apparent interfacial pH during cycling. During the first cycle, the working electrode was pressed against the ATR crystal, producing strong changes in phosphate band intensities and the extracted pH. For cycles 2 and 3, the electrode was lifted away from the ATR crystal, after which the phosphate bands and derived pH remained nearly constant.

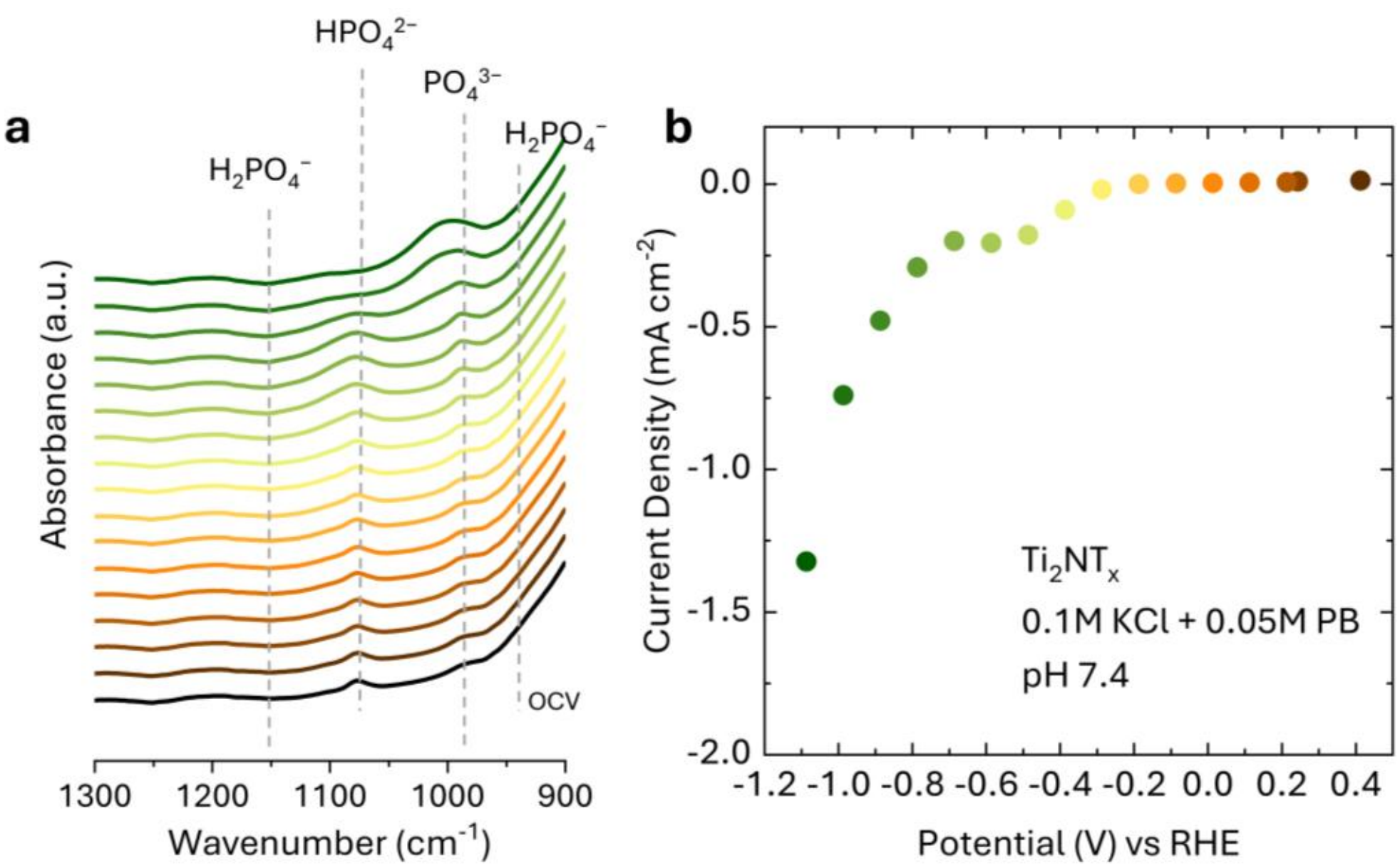

Figure S10. Potential-dependent phosphate response of Ti_2NT_x in 0.1 M KCl containing 0.05 M phosphate buffer at an initial pH of 7.4. (a) In situ ATR-FTIR spectra collected during staircase voltammetry. (b) Corresponding staircase voltammogram showing the potential-dependent current density. Because the $H_2PO_4^-$ contribution is weak at this phosphate concentration, quantitative interfacial pH values were not extracted.

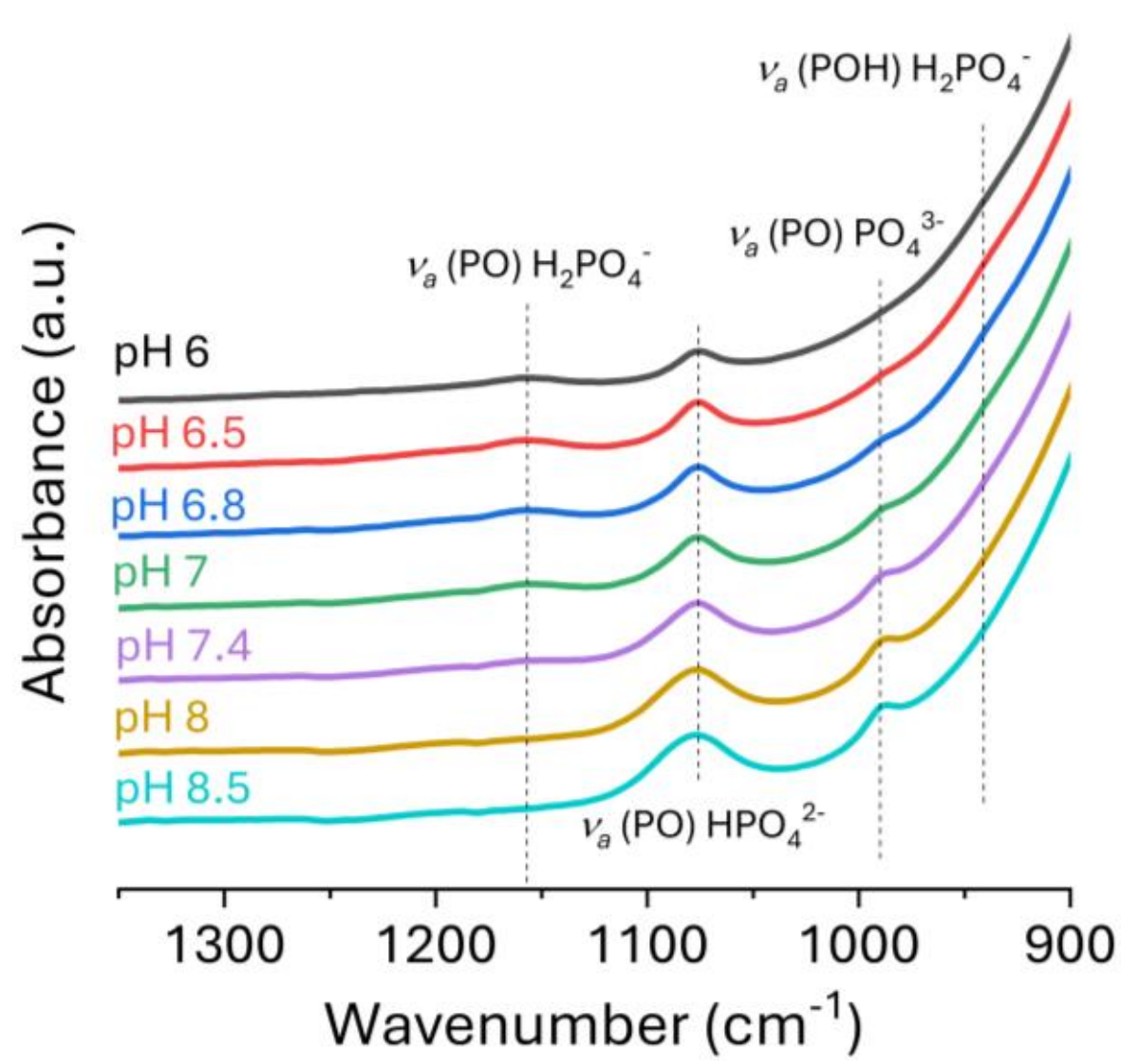

Figure S11. FTIR spectra of 0.05 M phosphate buffer in 0.1M KCl at different bulk pH values.

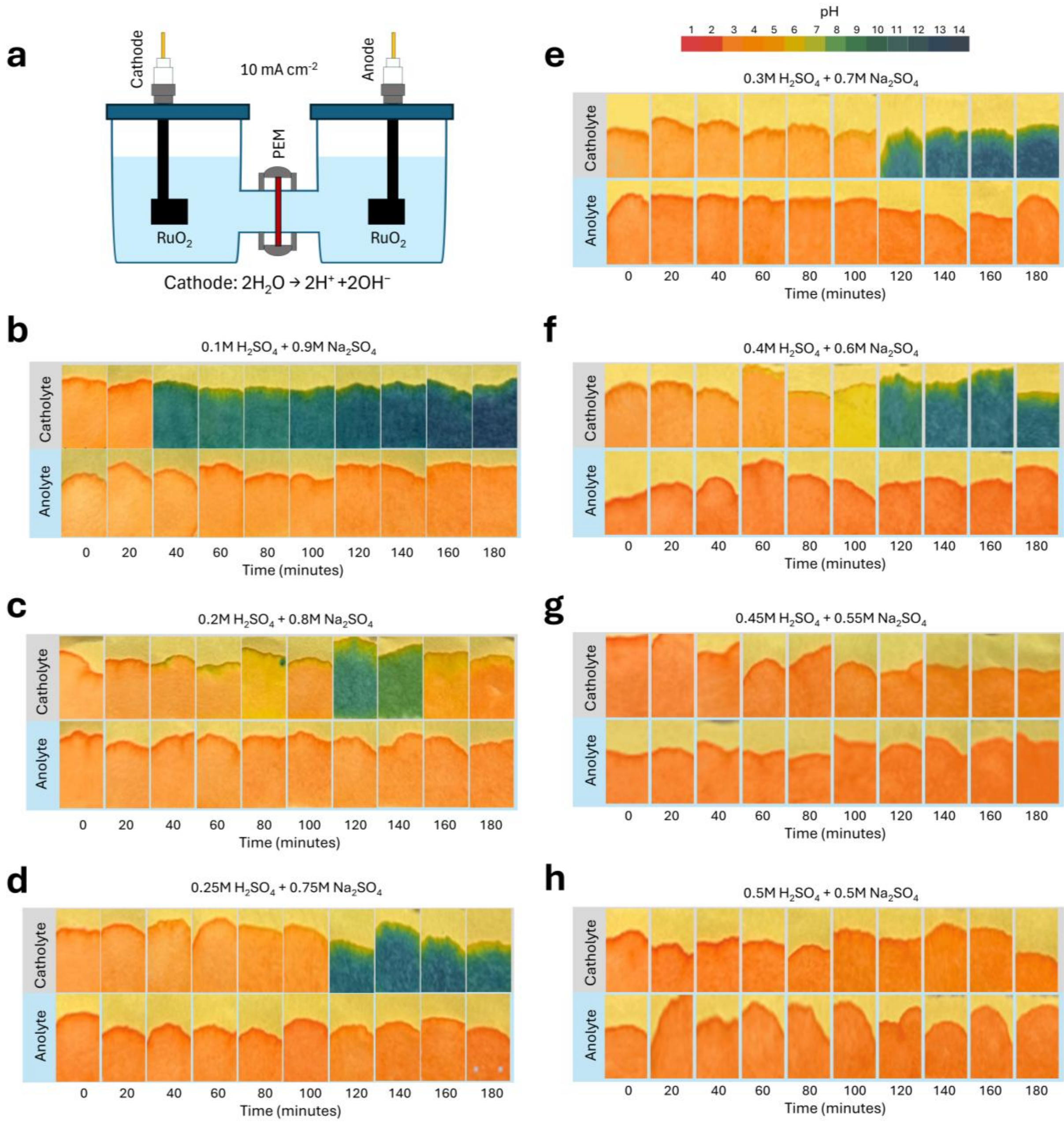

Figure S12. (a) Schematic of the H-cell electrolysis experiment used to visualize pH evolution under constant current operation. RuO_2 electrodes were used in both cathode and anode compartments separated by a PEM, and electrolysis was performed at 10 mA cm^{-2}. (b–h) Time-dependent colorimetric pH images of the catholyte and anolyte for mixed H_2SO_4/Na_2SO_4 electrolytes with increasing acid fraction.

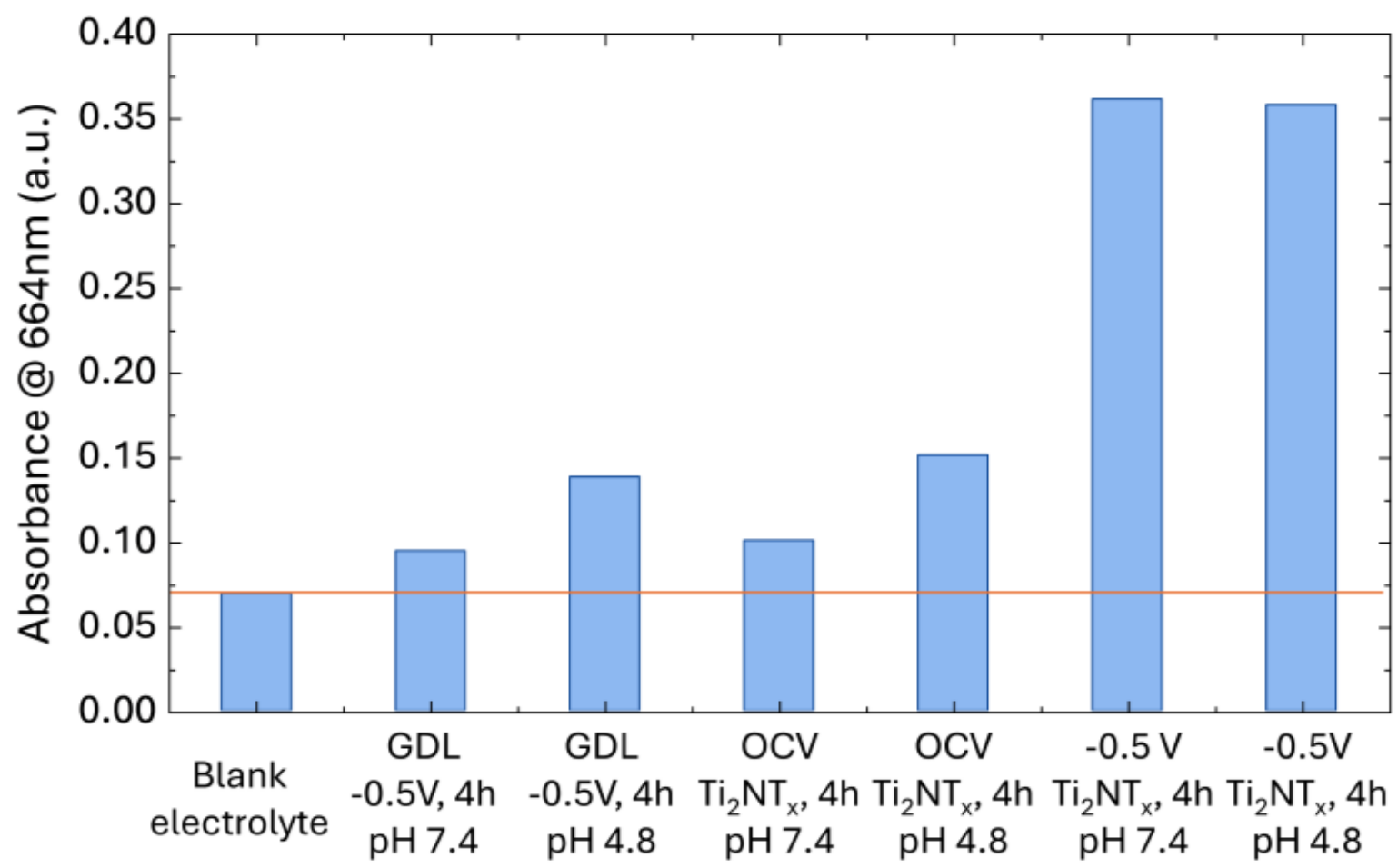

Figure S13. Absorbance at 664 nm from the Berthelot test is shown for the blank electrolyte, GDL-only electrodes polarized at -0.5 V vs RHE for 4 h, Ti_2NT_x electrodes held at open-circuit potential for 4 h, and Ti_2NT_x electrodes polarized at -0.5 V vs RHE for 4 h. Measurements were performed at initial pH values of 7.4 and 4.8 where indicated.

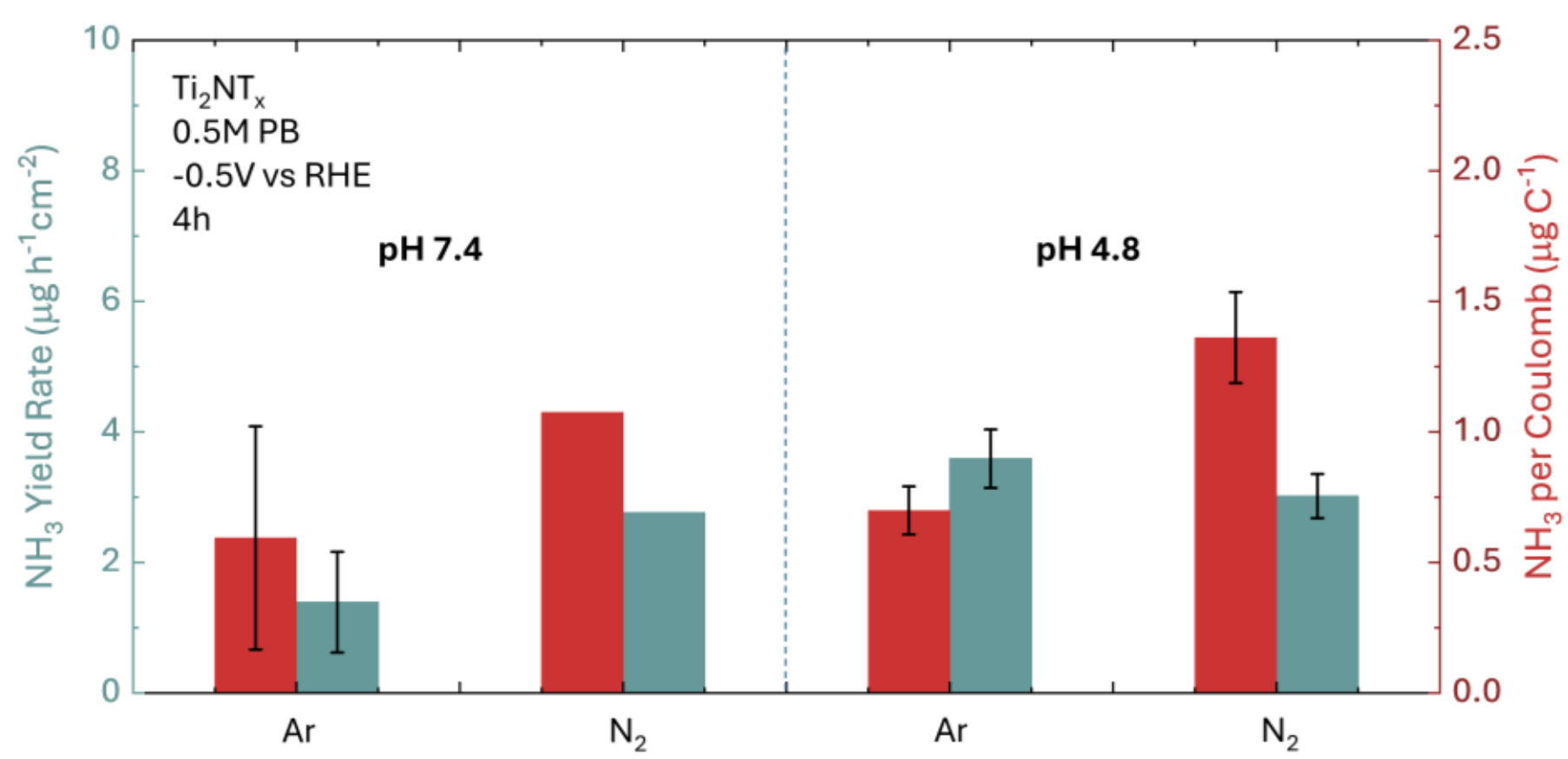

Figure S14. Effect of initial electrolyte pH on NH_3 formation from Ti_2NT_x in phosphate buffer. Electrolysis was performed for 4 h at -0.5 V vs RHE in 0.5 M phosphate buffer at initial pH values of 7.4 and 4.8 under Ar and N_2 atmospheres.
